# Potential Associations Among Alteration of Salivary miRNAs, Saliva Microbiome Structure, and Cognitive Impairments in Autistic Children

**DOI:** 10.3390/ijms21176203

**Published:** 2020-08-27

**Authors:** Marco Ragusa, Maria Santagati, Federica Mirabella, Giovanni Lauretta, Matilde Cirnigliaro, Duilia Brex, Cristina Barbagallo, Carla Noemi Domini, Mariangela Gulisano, Rita Barone, Laura Trovato, Salvatore Oliveri, Gino Mongelli, Ambra Spitale, Davide Barbagallo, Cinzia Di Pietro, Stefania Stefani, Renata Rizzo, Michele Purrello

**Affiliations:** 1 Department of Biomedical and Biotechnological Sciences, Section of Biology and Genetics G. Sichel, University of Catania, 95123 Catania, Italy; mragusa@unict.it (M.R.); mirabella.federica.91@gmail.com (F.M.); giovannilau91@hotmail.it (G.L.); matildecirnigliaro@gmail.com (M.C.); duiliabrex@gmail.com (D.B.); barbagallocristina@unict.it (C.B.); dbarbaga@unict.it (D.B.); dipietro@unict.it (C.D.P.); 2 Oasi Research Institute—IRCCS, 94018 Troina, Italy; 3 Department of Biomedical and Biotechnological Sciences, Section of Microbiology, University of Catania, 95123 Catania, Italy; m.santagati@unict.it (M.S.); ltrovato@unict.it (L.T.); oliveri@unict.it (S.O.); ginomongelli@unict.it (G.M.); ambra.spit@gmail.com (A.S.); stefanis@unict.it (S.S.); 4 Department of Clinical and Experimental Medicine, Section of Child and Adolescent Psychiatry, University of Catania, 95123 Catania, Italy; carlanoemidomini@gmail.com (C.N.D.); mariangelagulisano@gmail.com (M.G.); rita.barone@unict.it (R.B.); rerizzo@unict.it (R.R.); 5 Bio-nanotech Research and Innovation Tower (BRIT), University of Catania, 95123 Catania, Italy

**Keywords:** ASD, microRNA, oral microbiota, oral cavity, dysbiosis, correlations, Nanostring, Illumina, TaqMan assays

## Abstract

Recent evidence has demonstrated that salivary molecules, as well as bacterial populations, can be perturbed by several pathological conditions, including neuro-psychiatric diseases. This relationship between brain functionality and saliva composition could be exploited to unveil new pathological mechanisms of elusive diseases, such as Autistic Spectrum Disorder (ASD). We performed a combined approach of miRNA expression profiling by NanoString technology, followed by validation experiments in qPCR, and 16S rRNA microbiome analysis on saliva from 53 ASD and 27 neurologically unaffected control (NUC) children. MiR-29a-3p and miR-141-3p were upregulated, while miR-16-5p, let-7b-5p, and miR-451a were downregulated in ASD compared to NUCs. Microbiome analysis on the same subjects revealed that *Rothia, Filifactor, Actinobacillus*, *Weeksellaceae, Ralstonia, Pasteurellaceae,* and *Aggregatibacter* increased their abundance in ASD patients, while *Tannerella, Moryella* and *TM7-3* decreased. Variations of both miRNAs and microbes were statistically associated to different neuropsychological scores related to anomalies in social interaction and communication. Among miRNA/bacteria associations, the most relevant was the negative correlation between salivary miR-141-3p expression and *Tannerella* abundance. MiRNA and microbiome dysregulations found in the saliva of ASD children are potentially associated with cognitive impairments of the subjects. Furthermore, a potential cross-talking between circulating miRNAs and resident bacteria could occur in saliva of ASD.

## 1. Introduction

Autism Spectrum Disorder (ASD) is a heterogeneous group of complex neurodevelopmental disorders characterized by impaired social communication and the presence of stereotyped and repetitive behaviors [1]. The etiopathogenesis of ASD is still unclear but is believed to be the complex result of a combination of genetic, epigenetic and environmental factors [2,3,4]. ASD is genetically highly heterogeneous. Both inherited and de novo ASD-associated variants have been characterized in hundreds of genes [5,6,7,8,9,10,11,12]. Immune dysregulation and gastrointestinal abnormalities are of particular interest in the light of several papers reporting ASD-associated disturbances in the peripheral, enteric and neuro-immune systems [13,14]. This association of ASD with a great prevalence of immune and gastrointestinal dysregulations led to investigations on the gut microbiome of ASD patients, which is emerging as a key regulator of intestinal physiology, neuroimmunity, and host behavior. Intriguingly, reports on gnotobiotic animals and probiotic studies have shown that microbiome dysregulation can directly induce behavioral and neuropathological endophenotypes of human ASD [15,16,17,18,19]. The gut microbiota represents a barrier against the proliferation of pathogenic organisms [20], playing a key role in the functioning of the host immune system [21], modulating gene expression [22], and reducing inflammation [23]. Alterations of brain structure and function development are related to modifications of the gut microbial composition because the interactions between this and the Central Nervous System (CNS) are already established during fetal life and maintained in adults [24]. Despite several reports about gut microbiota dysbiosis in ASD patients, there is little consensus across independent studies on specific bacterial species that are similarly altered [25]. Moreover, whether microbiome alteration is caused by ASD symptoms or whether it contributes to the ASD gut phenotypes is still unclear. While mounting evidence suggests a key role for the gut microbiota in ASD, the etiopathogenetic contribution of microorganisms living in the oral cavity has not been satisfactorily explored. The oral cavity represents the major gateway of bacteria into the human body: it is a unique and complex habitat [26], which harbors approximately 700 predominant taxa [27]. In addition, biologically relevant interactions between the saliva microbiome and other microbiomes in the human body may occur [28]. Several studies have shown that saliva microbiome is dysregulated in patients affected by systemic diseases, such as liver cirrhosis, diabetes, rheumatoid arthritis, and cancer [29,30,31,32,33]. The oral microbiome is modulated in response to several intrinsic and extrinsic factors that may in turn affect brain functionality [34]. The oral cavity is considered an important source of alterations that manifest at distant body sites, including the neural system [27]. Indeed, some studies reported the effects of the oral microbiome on neural functions. Accordingly, oral dysbiosis has been associated with Parkinson’s disease, Alzheimer’s disease, multiple sclerosis, and migraine [35,36,37,38,39]. Saliva also represents an important reservoir of molecules, including proteins and microRNAs (miRNAs), associated with the pathological development of neurological diseases and brain dysfunctions [40,41,42,43,44,45,46]. Recently, fourteen miRNAs were found differentially expressed in the saliva of ASD patients and showed significant correlations with Vineland neurodevelopmental scores [47]. Based on these premises, we report the miRnome and microbiome analysis from saliva of ASD children with the aim to verify the existence of a molecular correlation between miRNA dysregulation and salivary dysbiosis and their contribution to ASD pathogenesis.

## 2. Results

### 2.1. Demographic and Neuropsychological Characteristics

We recruited a total of 115 children from various socio-economic contexts (age range: 4–8 years): 76 treatment-naïve patients affected by ASD (M:F 62:15), mean age 7 (SD ± 1.5), which were compared to 39 neurologically unaffected controls (NUC) (M:F 29:11), mean age 6.75 (SD ± 1.51). Demographic, neuropsychological and hematological characteristics of the clinical sample are shown in Table 1.

### 2.2. Salivary miRNA Expression Profiling 

**Figure 1:**
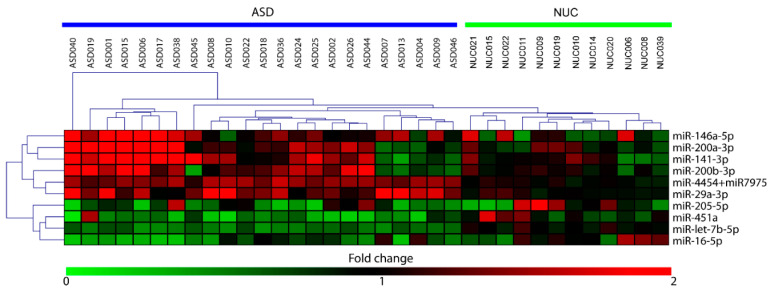
Heat-map of differentially expressed (DE)-miRNAs in saliva of Autistic Spectrum Disorder (ASD) and neurologically unaffected control (NUC) individuals. Heat-map of the miRNAs differentially expressed in saliva of ASD and NUC patients. The values of fold changes for each miRNA are color coded, as shown in the colored bar. The matrix was generated by plotting the fold changes calculated as the ratio between the normalized counts of each sample and the mean of normalized counts of all NUC samples. Sample clustering obtained through hierarchical clustering (Manhattan distance metric) approach is shown.

By using the nCounter NanoString technology we performed a high-throughput expression analysis of 800 microRNAs in saliva of 23 ASD patients and 12 NUC subjects. We identified 10 miRNAs as significantly differentially expressed (DE) in ASD patients compared to negative controls, six upregulated (miR-29a-3p, miR-141-3p, miR-146a-5p, miR-200a-3p, miR-200b-3p, miR-4454, and miR-7975) and four downregulated (miR-16-5p, miR-205-5p, miR-451a, and let-7b-5p). The relative expression of DE miRNAs is shown as heat-map in Figure 1.

### 2.3. Salivary miRNA Expression Validation

**Figure 2:**
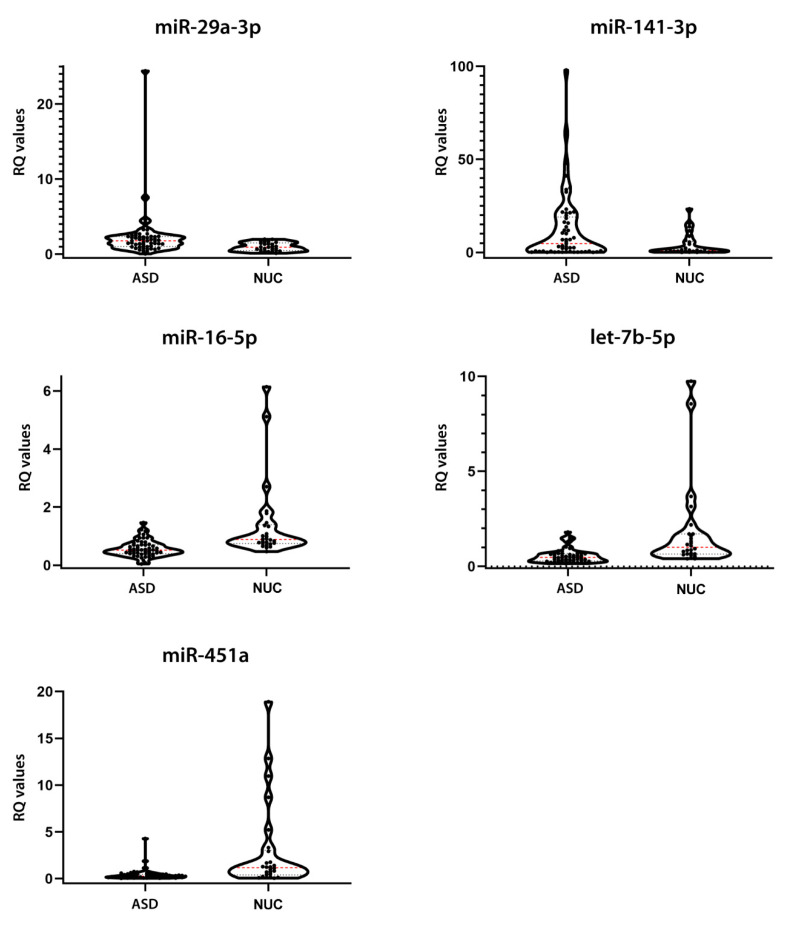
Expression validation of the 5 candidate salivary miRNAs. Violin plots of relative expression of the 5 miRNAs showing a statistically significant dysregulation in the validation group, assessed by Single TaqMan Assays: miR-29a-3p, miR-141-3p, miR-16-5p, let-7b-5p, and miR-451a. The black dots represent the samples; the dashed red line represents the median value; the dashed black line represents the quartiles.

To validate these findings, we tested the expression of the seven most dysregulated DE miRNAs (miR-16-5p, miR-29a-3p, miR-141-3p, miR-146a-5p, miR-200a-3p, miR-451a, and let-7b-5p) through single TaqMan assays in an independent cohort, composed of 53 ASD patients and 27 NUC subjects. Real Time-polymerase chain reactions (RT-PCR) results showed that five salivary miRNAs were altered in ASD patients compared to NUCs in a statistically significant manner. More specifically, miR-29a-3p (*p* = 0.0123, Cliff’s δ = 0.341) and miR-141-3p (*p* = 0.0431, Cliff’s δ = 0.277) were upregulated, while miR-16-5p (*p* = 0.0002, Cliff’s δ = −0.502), miR-451a (*p* = < 0.0001, Cliff’s δ = −0.520), and let-7b-5p (*p* = 0.0002, Cliff’s δ = −0.499) were downregulated in the validation group, confirming the results obtained by NanoString analysis. Although the expression of miR-146a-5p and miR-200a-3p evaluated by RT-PCR had the same dysregulation trend observed by NanoString profiling, these alterations were not statistically significant. Table 2 and Figure 2 show the relative expressions of the five DE miRNAs.

Salivary miRNA expression validation in ASD and NUC groups. *p*-values were obtained from Mann–Whitney test. Expression FC (Fold Change) values are shown.

The expression values of ASD and NUC are quite heterogeneous, similar to the results of NanoString profiling. Notwithstanding this endogenous variability, their expression differences resulted statistically significant. Cliff’s delta effect size values ranged from −0.5206 to 0.3417, showing small and medium effect sizes according to Cliff’s statistic, likely due to the complex heterogeneity of autism and the limited sample size.

### 2.4. Microbial Structure of the Saliva Microbiome in Children with ASD and NUCs

To evaluate changes in the microbial composition of the salivary microbiome, a total of 53 ASD and 27 NUC samples were sequenced using the Illumina MiSeq platform.

**Figure 3:**
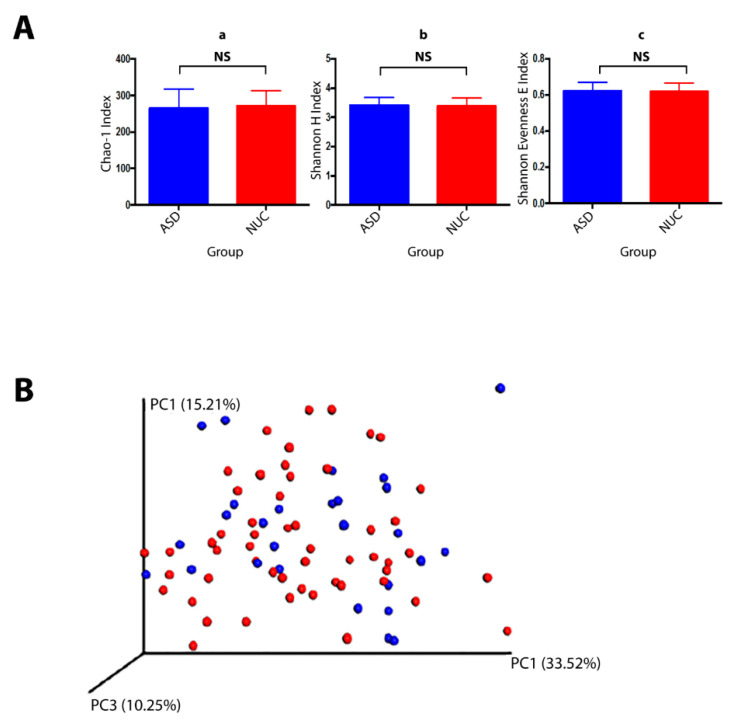
Bacterial community structures of the salivary microbiome in children with ASD and NUC groups. (**A**) Structural comparison of α-diversity of the salivary microbiome. Sequences were randomly subsampled at the rarefaction point (74,469) from dataset. Chao-1 index (a, community richness), Shannon H index (b, diversity), Shannon E index (c, evenness) were calculated for saliva samples. The bars depict mean ± SD of relative abundance rates. NS, *p* > 0.05. ASD (salivary samples *n* = 53), NUC (salivary samples collected from healthy controls, *n* = 27). (**B**) β-diversity. PCoA plot generated using weighted UniFrac distances shows none differences between the two groups (ASD in red and NUC in blue).

A total of 10,919,413 valid reads with an average of 127,464 reads per sample were generated (74,469–1,641,234 range). All sequences were assigned to 341 Operational taxonomic Units (OTUs) with at least 97% similarity level showing 10 phyla, 65 genera, and 86 species. The alpha diversity, Chao1 index (richness), Shannon index (diversity), and Shannon Evenness E index (evenness) revealed no significant differences between ASD and NUC groups (*p* > 0.05) although the Chao1 index showed a slight decrease in the ASD group (Figure 3A). The overall dissimilarities of microbial community structure between the two groups were calculated by using the weighted UniFrac distance. The beta diversity shown by PCoA (principal coordinate analysis) plot on weighted (accounting for the abundance of OTUs) revealed no clustering differentiation of the bacterial communities in the two groups (Figure 3B). The composition of the salivary microbiome between ASD and NUC was explored in terms of the relative abundances at different taxonomic levels through White’s non-parametric *t*-test; *p*-value < 0.05 by STAMP software. Predominant phyla in ASD and NUC groups were *Firmicutes* (41.1% vs. 42.7%), *Bacteroidetes* (18.9% vs. 22.3%), *Proteobacteria* (16.9% vs. 10.8%), *Actinobacteria* (14.1% vs. 13.3%), and *Fusobacteria* (7.6% vs. 9.3%) constituting 98.6% of salivary microbes (see Supplementary Figure S1). Overall, *Proteobacteria* were more abundant in ASD patients compare to control participants and a slight increase was also observed in Actinobacteria, while the other phyla were more abundant in the NUC group.

**Figure 4:**
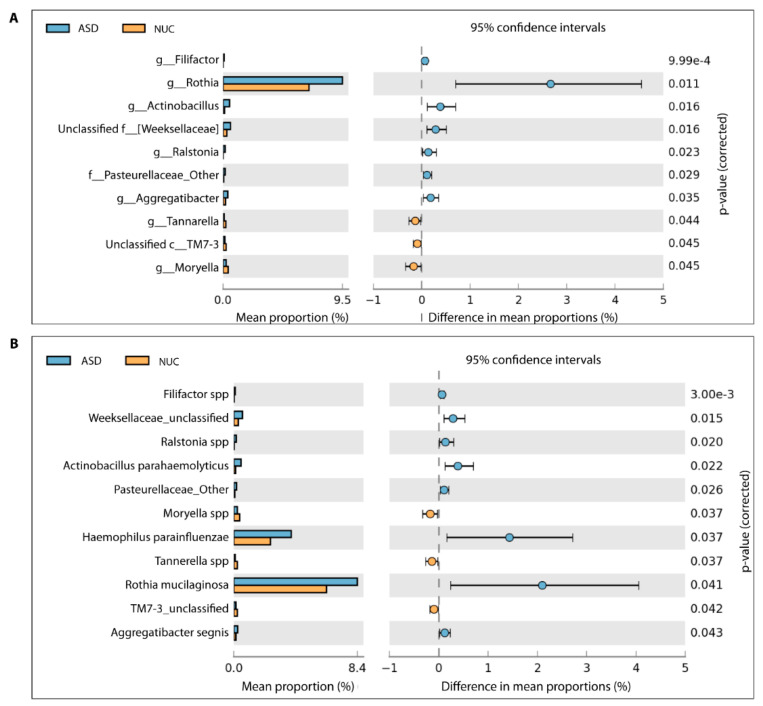
Different bacterial abundance in saliva of ASD and NUC groups. Statistical analysis of the bacterial abundance at genus (**A**) and species level (**B**) in ASD and NUC groups by applying a two-sided White’s non-parametric *t*-test.

A total of 65 genera were detected (see Supplementary Figure S2) and among these, 10 genera showed a statistically significant difference in relative abundance between ASD and NC children by applying the two-sided White’s non-parametric *t*-test (Figure 4A), and also confirmed by applying the Mann–Whitney–Kruskal–Wallis test and *t*-test/ANOVA ( Supplementary Table S1).

In particular, *Rothia*, *Filifactor*, *Actinobacillus*, *Weeksellaceae*, *Ralstonia*, *Pasteurellaceae*, and *Aggregatibacter* increased their abundance rates in ASD, while *Tannerella*, *Moryella*, and *TM7-3* decreased (Figure 4A). At the species level, we found statistically significant differences for 11 taxa out of 86 species (Figure 4B and Supplementary Figure S3). More specifically, *Filifactor* (Cliff’s δ = 0.261), *Weeksellaceae* (Cliff’s δ = 0.096), *Ralstonia* (Cliff’s δ = 0.217), *Actinobacillus parahaemolyticus* (Cliff’s δ = 0.077), *Pasteurellaceae* (Cliff’s δ = 0.043), *Haemophilus parainfluenzae* (Cliff’s δ = 0.220), *Rothia mucilaginosa* (Cliff’s δ = 0.078), and *Aggregatibacter segnis* (Cliff’s δ = 0.032) were more abundant in saliva of the ASD group, in contrast to *Moryella* (Cliff’s δ = −0.400), *Tannerella* (Cliff’s δ = −0.412), and *TM7-3* (Cliff’s δ = −0.455) (Figure 4B). Cliff’s delta effect size values ranged between −0.4556 and 0.2613 and, according to the Cliff’s statistic, could be considered small and mostly moderate.

### 2.5. Correlation and Negative Binomial Regression Analyses Among Salivary miRNAs, Bacteria, and Neuropsychological/Hematological Parameters

**Figure 5:**
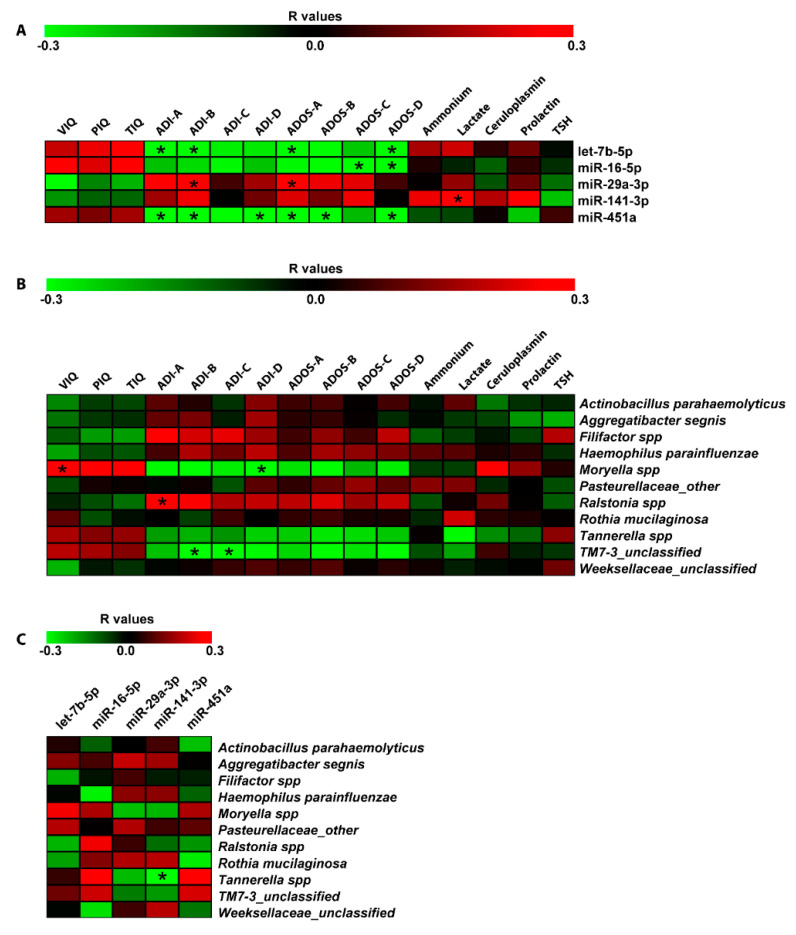
Correlation analysis of miRNA expression, microbiome Operational taxonomic Units (OTUs), neuropsychiatric/metabolic parameters in saliva. Correlation matrices built by calculating spearman correlation coefficients for (**A**) miRNA expression and neuropsychiatric/metabolic parameters; (**B**) microbiome OTUs and neuropsychiatric/metabolic parameters; and (**C**) microbiome OTUs and miRNA expression. The correlation coefficient is indicated by a color gradient from green (negative correlation) to red (positive correlation), as shown in the colored bar. Statistically significant *p*-values corrected for multiple comparisons by using Bonferroni–Šídák approach are indicated by asterisks. VIQ: Verbal Intelligence Quotient; PIQ: performance Intelligence Quotient, TIQ: Total Intelligence Quotient; ADOS-A: Communication; ADOS-B: Social Interaction; ADOS-C: Imagination; ADOS-D: Repetitive and Restricted Behavior; ADI-A: Qualitative anomalies in social interaction; ADI-B: Qualitative anomalies in communication; ADI-C: Repetitive and restricted behavior; ADI-D: Anomalies in neurodevelopment arisen before 36 months old.

Spearman correlation analyses were computed among DE miRNAs, bacteria differentiating ASD and NUCs and neuropsychological/hematological scores (Figure 5). This statistical analysis revealed a negative relationship between let-7b-5p, miR-451a, and ADI-A (Qualitative anomalies in social interaction) (*r* = −0.37; *r* = −0.38, respectively to let-7b-5p and miR-451a), ADI-B (Qualitative anomalies in communication) (*r* = −0.33, *r* = −0.36, respectively), Autism Diagnostic Observation Schedule (ADOS)-A (Communication) (*r* = −0.32, *r* = −0.39, respectively), ADOS-D (Repetitive and Restricted Behavior) (*r* = −0.38, *r* = −0.36, respectively). MiR-451a showed a negative correlation with ADI-D (Anomalies in neurodevelopment arising before 36 months old) and ADOS-B (Social Interaction) (*r* = −0.34, *r* = −0.40, respectively). MiR-16-5p was negatively related to ADOS-C (Imagination) (*r* = −0.37) and ADOS-D (*r* = −0.36); while a positive correlation was found between miR-29a-3p and ADI-B (*r* = 0.37) and ADOS-A (*r* = 0.33). Finally, miR-141-3p was the only miRNA to have a linear correlation with a metabolic parameter: positive association with lactate (*r* = 0.38) (Figure 5A).

The same correlation analysis was also performed on salivary bacteria. We found a positive correlation between *Moryella* and VIQ (*r* = 0.32), and a negative relationship between *Moryella* and ADI-D (*r* = −0.34). *TM7-3* exhibited a negative relationship with ADI-B (*r* = −0.37) and ADI-C (*r* = −0.34); while, *Ralstonia* showed a positive correlation with ADI-A (*r* = 0.34) (Figure 5B). Moreover, computing the Spearman correlations between salivary DE miRNAs and bacteria, we detected a negative association between *Tannerella* and miR-141-3p (*r* = −0.30) (Figure 5C).

**Figure 6:**
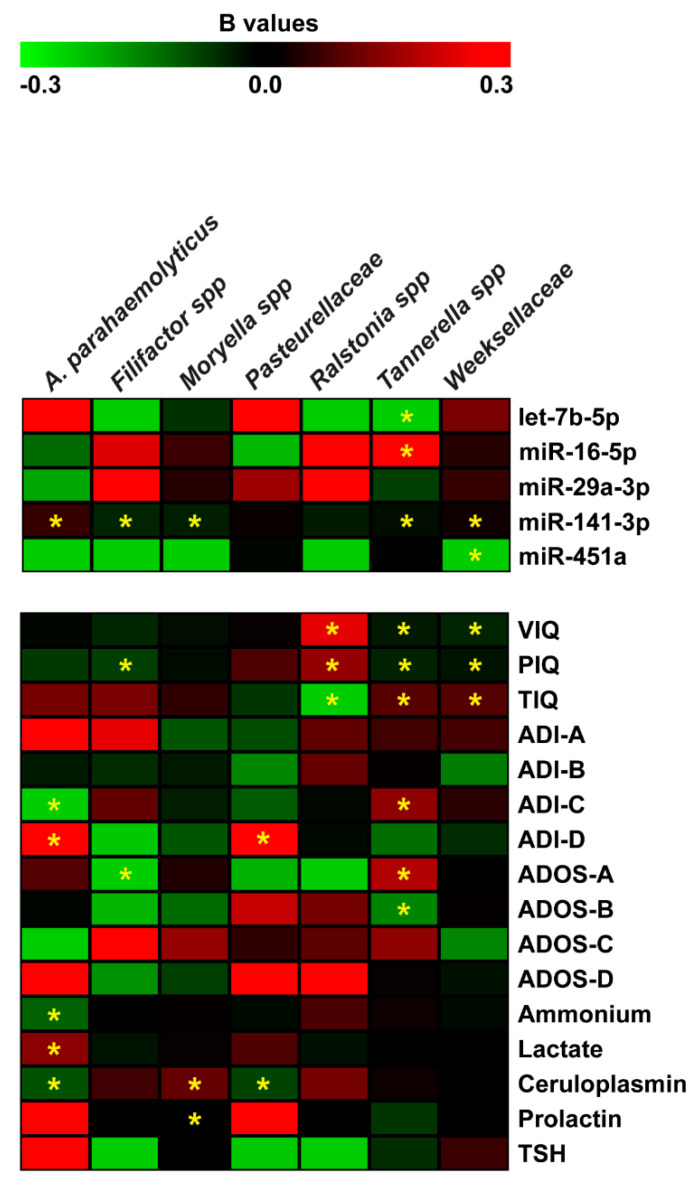
Negative binomial regression analysis between miRNA expression, neuropsychiatric/metabolic parameters and microbiome abundance. Coefficient regression matrix from negative binomial regression model predicting abundances of microbiome species. The regression coefficient is indicated by a color gradient from green (negative prediction) to red (positive prediction), as shown in the colored bar. Statistically significant *p*-values are indicated by asterisks.

For microbiome data, we also applied the negative binomial regression. The Negative Binomial (NB) model was positively assessed for seven (*Tannerella*, *Weeksellaceae*, *Moryella*, *Filifactor*, *Ralstonia*, *Pasteurellaceae*, and *Actinobacillus parahaemolyticus*) of the eleven microbiome taxa and revealed many statistically significant regressions shown in Figure 6. For each of the predictor variable, Supplementary Table S2 reports a regression coefficient (B) with a *p*-value (*p* < 0.05) resulting from the Wald Chi-Square test. Effect size measures, reported as Standardized mean difference (SMD) values, showed small and moderate effects (see Supplementary Table S2). Negative binomial regression (NBR) results were partially confirmed applying the multiple and simple linear regression models. These additional analyses confirmed the regression between Total Intelligence Quotient (TIQ), ADOS-A, ADOS-B and *Tannerella*; miR-141-3p and *Weeksellaceae*; miR-141-3p, ceruloplasmin and *Moryella*; PIQ and *Filifactor*; miR-141-3p, ADI-C, ADI-D, ammonium and *Actinobacillus parahaemolyticus* (see Supplementary Table S3).

### 2.6. Functional Enrichment Analyses

**Figure 7:**
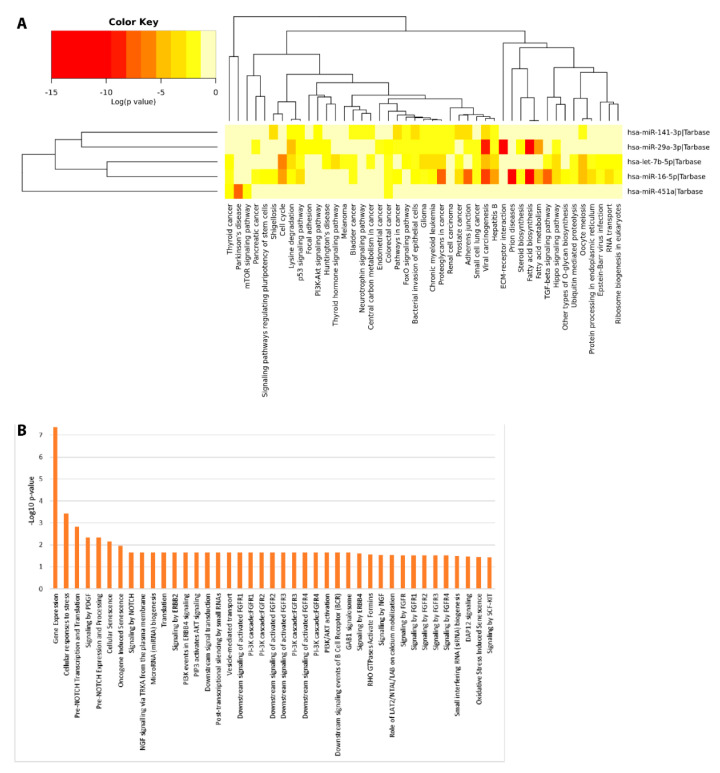
Functional Enrichment analysis of DE miRNAs. Functional enrichment analysis of miRNA targets within KEGG (Kyoto Encyclopedia of Genes and Genomes) pathways (**A**) and Reactome database (**B**) by DIANA-mirPath v.3 web server and miRNet tool, respectively.

Pathway enrichment analyses were computed to investigate the potential biological impact associated with the differential expression of the miRNAs reported in this study. We computationally identified a list of statistically over-represented gene ontologies and biological pathways (Figure 7) potentially related to ASD, as Pre-NOTCH transcription and Translation (False Discovery Rate (FDR) corrected *p* = 0.0015) and Pre-NOTCH Expression and Processing signaling pathway (FDR corrected *p* = 0.00471), signaling by NOTCH (FDR corrected *p* = 0.0221), signaling by NGF (FDR corrected *p* = 0.0291) through which cell fate decisions in neuronal development are regulated. Moreover, we found Parkinson’s disease related pathways (FDR corrected *p* = 0.0002), involved in synaptic and mitochondrial dysfunction and neuroinflammation; Hippo signaling pathway (FDR corrected *p* = 3.991× 10^–6^), FoxO (*Forkhead box O*) signaling pathway (FDR corrected *p* = 0.0001), PI3K-Akt signaling pathway (FDR corrected *p* = 0.0008), and mTOR signaling pathway (FDR corrected *p* = 0.02), involved in many biological events such as apoptosis, cellular stress and cell-cycle control. In addition, bacterial invasion of epithelial cell (FDR corrected *p* = 1.19 × 10^–6^) related pathways were also found. These data would represent an interesting link between the differential expression of miRNAs and the bacterial abundance alterations found in saliva.

These computational data would suggest a functional involvement of DE salivary miRNAs in molecular signaling pathways related to the development of cognitive functions, often reported to be dysfunctional in ASD. This observation is particularly intriguing because it hints at the possibility that some molecular alterations of ASD neuronal circuits could be mirrored in saliva by means of differential secretion of miRNAs.

## 3. Discussion

### 3.1. Circulating miRNAs and Microbiome Structure are Altered in Saliva of Pediatric ASD Patients

Recent studies have shown that perturbations in the normal structure of microbiome have a close relationship with human health and disease [19,31,32] and there is mounting evidence that alterations of the GI microbiome may influence neurological disorders and autistic behavior [48]. The shift of microbial communities in the oral cavity in these diseases is being increasingly studied to look at possible early diagnostic markers [25,35,36,39]. Several studies have revealed that miRNAs play very important roles in neural developmental processes [49,50,51,52,53], resulting in neuropsychiatric disorders [54,55] and neurodegenerative diseases [56,57], although their exact pathophysiologic role remains unclear [58]. In the present study, the salivary miRNA content and the oral microbiome were evaluated in a group of pediatric patients with ASD syndrome, compared with neurotypical subjects. MiRnome and microbiome dysregulation in saliva of ASD patients was previously reported by other groups, but there is only a partial concordance with the data presented here [47,59,60,61]. The salivary microbiome structure in human beings is strongly affected by the geographical, climatic and ethnic origin of samples [62,63,64]. This could be due to the different ratio of dietary protein/ carbohydrate intake that, in turn, modulates salivary pH [65]. This biochemical variability, such as the inconstancy of desquamated oral epithelial cells in saliva among people, would lead to a heterogeneous population of miRNAs in saliva among individuals [66].

In the present paper, a higher relative abundance of *Proteobacteria* and *Actinobacteria* phylotypes and a lower amount of *Bacteroidetes*, *Firmicutes,* and *Fusobacteria* were found in ASD patients. In addition, an increased abundance of *Rothia mucilaginosa, Filifactor, Actinobacillus parahaemolyticus. parahaemolyticus*, *Weeksellaceae, Ralstonia, Pasteurellaceae*, *Agregatibacter segnis*, and *Haemophilus parainfluenzae,* as well as a reduced amount of *Tannerella, Moryella* and *TM7-3* were observed in the saliva of ASD patients. These observations could contribute to define the dysbiotic signatures of disease. In particular, a similar trend for *Haemophilus* and *Rothi*a is supported by a previous observation [60].These taxa are often involved in several diseases, especially in immunocompromised hosts [67,68]. In the present study, many groups of microorganisms differentially abundant in ASD children were previously associated with CNS (Central Nervous System) disorders, such as Parkinson’s and Alzheimer’s Disease [69,70]. In some cases, these microorganisms are also relevant pathogens of periodontal diseases. It is noteworthy that *Filifactor* and *Tannerella* were previously reported as strong indicators of dysbiosis [71,72].

Previous papers reported that the five DE miRNAs we identified in ASD saliva were associated to neurological diseases, including ASD, in cellular and extra-cellular contexts. In Alzheimer’s patients, miR-29a was upregulated in cerebrospinal fluid (CSF) [73], whereas, miR-451a and miR-16-5p were downregulated in exosomes from CSF of young-onset (YOAD) subjects [74] and miR-141 decreased in the plasma fraction enriched in exosomes [75]. Moreover, the levels of miR-29a-3p were decreased in whole blood [76] and those of miR-141-3p in serum of Parkinson’s patients [77]. Levels of let-7b-5p were decreased in the saliva of children with mild traumatic brain injury [78]. Intriguingly, a downregulation of miR-451a and miR-16-5p was also identified in peripheral blood of ASD children [79]. Furthermore, miR-451a and miR-16-5p were upregulated and downregulated, respectively, in lymphoblastoid cell lines from autistic monozygotic twins [80]. Finally, miR-451 also showed an increased abundance in post-mortem ASD brain [81]. These findings would suggest a critical role for these miRNAs in the development of cognitive functions. Molecular pathways identified in our functional enrichment analysis would support the involvement of these miRNAs in ASD related mechanisms. Dysregulation of IGF-I/PI3K/AKT/mTOR signaling is associated with ASD pathogenesis by affecting the myelination, synaptic plasticity, mechanisms of social interactions, learning and immune functions [10,82,83]. The Hippo signaling pathway is related to neuropsychiatric disorders such as schizophrenia, bipolar disorder, obsessive-compulsive disorder and ASD, playing an important role in neural development and neuronal maintenance [84]. Finally, NOTCH, FoxO, and NGF pathways play multiple roles in neurodevelopment in the CNS regulating neurogenesis, synaptic plasticity, learning, memory, and behavior [85,86,87].

### 3.2. Potential Associations Among Cognitive Impairments, Salivary miRNA Expression and Microbiome Alteration in ASD Children

We found several linear associations between salivary miRNA expression and anomalies of social interaction and communication, expecially for let-7b, miR-451a, and miR-29a-3p (Figure 5A). Impairments in social-emotional reciprocity and relationships, verbal and nonverbal communicative behavior, cognitive skills, lead to increase the risk of social isolation and rejection of ASD children in daily social contexts. Some studies reported an association between social isolation and consequent psychological stress and miRNA dysregulation. For instance, miR-141-3p expression progressively increases in a time-dependent manner in mouse models of post-stroke social isolation [88], and members of the miR-29 family increased their expression during the healing process of oral palatal mucosal wounds [89]. Moreover, decreasing plasmatic let-7b-5p correlated strongly with cognitive impairment in the presence of severe alcohol use disorder [90] and it was also related to high stress conditions [91]. Levels of circulating miR-451a in the blood of depressed patients were found reduced and negatively associated with the Hamilton Depression Scale that is used to rate the severity of depression: a depression-like phenotype is often observed in autistic patients [92,93].

In blood of ASD patients elevated levels of lactate and its synthesizing enzyme, lactate dehydrogenase (LDH), reflect the mitochondrial energy metabolism dysfunctions [94]. Interestingly, miR-141-3p was found to induce mitochondrial dysfunctions in obese mice by inhibiting PTEN [95]. Moreover, circulating miR-141-3p positively correlated with LDH levels in rectal cancer [96], as well as, miR-141 positively regulated expression of LDH by inhibiting MAP4K4 in breast cancer [97]. These observations corroborate the positive correlation we found between miR-141 and lactate and suggest that it could represent a combined extracellular phenotype of ASD metabolic abnormalities.

*Moryella* showed a signature that negatively correlated with anomalies in neurodevelopment arising before the age of 36 months (ADI-D); the same *genus* was also linked to the Verbal Intelligence Quotient. *Ralstonia* positively correlated with a worst Qualitative anomaly in social interaction (ADI-A). Finally, the low abundance of phylotype TM7-3, often associated with human inflammatory mucosal diseases [98], negatively correlated with the increasing of both Qualitative anomalies in communication (ADI-B) and Repetitive and restricted behavior (ADI-C).

The negative binomial regression analysis revealed that the abundance of seven species was significantly related to cognitive impairments, especially for *Tannerella* [99] (Figure 6). In particular, VIQ, PIQ, and TIQ together with behavior (ADI-C), speech and communication anomalies (ADOS-A) were significant predictors of *Tannerella* abundance. In accordance with these results, it has been reported that periodontal disease is related to cognitive decline [100], disability, speech and communication impairment, low self-esteem and quality of life [101]. Interestingly, the regression analyses uncovered a significant relationship among all the Intelligence Quotients (i.e., VIQ, PIQ, and TIQ) and *Weeksellaceae* and *Ralstonia* abundances.

These results pave the way to suggestion that miRNA expression and microbiome structure alteration could be a consequence of ASD symptomatology.

### 3.3. Potential Crosstalk between miRNAs and the Microbiome in Saliva

It is known that microbiota can secrete bioactive compounds able to modify the host epigenome; as well as, miRNAs from the host could selectively regulate the functions of microbiota.

Linear correlation analysis between miRNA expression and microbiome data in saliva led to the identification of a negative relationship between miR-141-3p and *Tannerella.* This inverse correlation has already been reported. It has been demonstrated that miR-141-3p was underexpressed in the gingiva of patients affected by periodontal disease compared to healthy gingival tissue [102], while, *Tannerella* is one of the major Gram-negative periodontal pathogens and it is already identified as a marker of autism [60,99]. The negative binomial regression sheds a light to other considerations regarding the potential interaction between: (i) miRNAs and neuropsychological parameters (as predictors) and (ii) microbiome (as outcome variables) in terms of expected change in the outcome variable for a one-unit change in the predictor variable. For example, NB regression revealed new potential interactions between the miRNAs previously associated with cognitive impairments (based on linear correlation analyses) and specific bacteria: let-7b-5p and miR-16-5p with *Tannerella*; miR-451a and *Weeksellaceae;* miR-141-3p and *Tannerella*, *Weeksellaceae*, *Moryella, Filifactor* (Figure 6). MiR-141-3p is known to be expressed in intestinal epithelial cells and a potential biomarker for gut dysbiosis [103].

Although this issue is quite unexplored in the literature, recent reports demonstrated that fecal miRNAs could contribute to shaping the composition of the gut microbiome [104,105], suggesting a mechanism by which host cells can regulate the microbial community.

The significant relationships found in the regression analysis may suggest that the potential crosstalk between oral bacteria and salivary miRNAs could be based on a general response of host to dysbiosis involving several miRNA families, as well as bacterial phyla [106,107,108,109,110,111,112,113].

Taken together, all these findings suggest that in the saliva of autistic children quantitative differences in miRNA expression and bacteria abundance are present and potentially associated with anomalies in social interaction and communication.

## 4. Materials and Methods

### 4.1. Ethics Approval and Consent to Participate

All experiments were approved by the local Ethics Committee, Comitato Etico Catania 1, University of Catania (ID: 002430-36) prior to sample collection. Written, informed consent was obtained from all parents and each participant who were able gave their informed assent.

All experimental methods were in accordance with Helsinki Declaration.

### 4.2. Participant Selection

From a database of more than 2000 patients, 76 treatment-naïve patients affected by ASD were recruited and studied from January to October 2018 in the outpatient service of the Child and Adolescent Psychiatry Unit (Department of Clinical and Experimental Medicine, University Hospital of Catania).

The inclusion criteria were clinical diagnosis of ASD, according to the criteria of the Diagnostic and Statistical Manual of Mental Disorders, Fifth Edition (DSM-5, APA 2013), and the absence of other medical, neurological, genetic or metabolic condition such as epilepsy, cytogenetically visible chromosomal abnormalities, copy number variants (CNVs) or single-gene disorders. They were compared to 39 neurologically unaffected controls (NUC), without any history of ASD and who suffered from neither chronic neurological, metabolic or genetic diseases nor psychiatric disorders. All participants to the study were Caucasians from Sicily, randomly recruited from various socio-economic contexts.

The entire cohort was split into 2 sets of samples: (a) discovery set, composed by 23 ASD and 12 NUC; (b) validation set, made by 53 ASD and 27 NUC. The discovery set was used to perform expression profiling by NanoString technology, while the validation set was analyzed by real time PCR single assays and 16S rRNA microbiome screening.

### 4.3. Assessment

All participants were assessed by a child and adolescent neuropsychiatrist expert in ASD (RR) with the following instruments: WISC-III (Wechsler Intelligence Scale for Children, III edition) [114] or WPSSI (Wechsler Preschool and Primary Scale of Intelligence) [115] as an evaluation of both IQ (Intelligence Quotient) and cognitive functioning, ADOS (Autism Diagnostic Observation Schedule) and ADI-R (Autism Diagnostic Interview–Revised) to evaluate ASD symptoms.

Based on these scales and schedules, the assessment procedure was carefully conducted assigning specific scores which provided a measure of autism severity. Neuropsychological features of participants are summarized in Table 1.

### 4.4. Sample Collection

All participants were instructed to refrain from eating or drinking for at least 3 h prior to saliva collection. Saliva samples were collected in a good status of oral hygiene (i.e., brushing teeth once/twice daily), without any tooth decay. Saliva collection was always performed between 8:30 and 10:30 a.m. to avoid any potential microbiome and miRnome oscillation due to the circadian rhythms. From a minimum of 800 μL to a maximum of 4 mL of non-stimulated and naturally outflowed saliva was collected into 50 mL conical centrifuge tubes. Saliva samples were centrifuged at 10,000 rpm for 15 min at 4 °C to separate the pellet and supernatant for microbiological analysis and miRNA expression assays, respectively. The pellets were immediately processed, while supernatants were aliquoted into 2 mL RNase-free tubes and stored at −80 °C until analysis.

To test the hematological parameters of each study participant, all participants were instructed to refrain from eating or drinking for at least 3 h prior to blood collection, which was performed between 8:30 and 10:30 a.m. Peripheral blood samples were collected through a butterfly device into a 5 mL collection tube. Collection tubes were treated according to current and standard procedures for clinical samples.

### 4.5. RNA Extraction

Extraction of total RNA was carried out from 800 μL of saliva samples using Qiagen miRNeasy Mini Kit (Qiagen, GmbH, Hilden, Germany), according to Qiagen Supplementary Protocol for purification of RNA (including small RNAs) from serum or plasma [46,116]. RNA was eluted in 200 μL RNAse-free water and then precipitated by adding 20 μg glycogen, 0.1 volumes 3 M sodium acetate and 2.5 volumes ice-cold 100% ethanol. After incubation at −80 °C overnight, RNA was centrifuged and washed twice in ice-cold 75% ethanol and resuspended in 7 μL RNAse-free water. The yield and quality of the RNA samples were assessed by using NanoDrop Lite Spectrophotometer (Thermo Fisher Scientific, Wilmington, DE, USA).

### 4.6. MiRNA Profiling by NanoString Technology

To profile the expression of circulating miRNAs from saliva, the NanoString nCounter system assays were performed using the NanoString platform and the nCounter Human v3 miRNA Expression Assay Kits (NanoString Technologies, Seattle, WA, USA), according to the manufacturer’s instructions. MiRNA profiling was performed on 23 ASD patients and 12 NUCs, starting from 3 μL of isolated RNA (approximately 150 ng). Samples were processed using the automated nCounter Prep Station; following hybridization, they were purified and immobilized on a sample cartridge for quantification and data collection by using the nCounter Digital Analyzer. The nSolver 3.0 software was used for data analysis. The endogenous control was selected from the arrays in a similar way to the GMN (global median normalization) method [55,117]. By this approach, we identified miR-21-5p as the best endogenous control for our experimental model.

### 4.7. MiRNA Data Validation by Single TaqMan Assays

MiRNAs found differentially expressed by NanoString analysis were assessed in a larger independent cohort of 53 ASD patients and 27 NUCs. Validation analysis was performed on 20 ng of salivary RNA by using single TaqMan MicroRNA Assays (Applied Biosystems, Foster City, CA, USA) in a 7900HT Fast Real-Time PCR System (Applied Biosystems), according to the manufacturer’s instructions. Data analysis was computed on SDS v2.4 and RQ Manager 1.2.1 (Applied Biosystems). Expression fold change values are shown as geometric mean of relative quantification (RQ) values obtained by applying the 2^−∆∆*C*t^ method [118].

### 4.8. DNA Extraction, 16S rRNA Gene Library Preparation, and Sequencing

DNA from salivary samples was extracted with the PureLink Genomic DNA Kit (Thermo Fisher Scientific, Waltham, MA, USA) according to the manufacturer’s instructions. Extracted DNAs were checked for quality and quantity by NanoDrop2000 Spectrophotometer (Thermo Fisher Scientific, USA). A negative control only containing the buffer was included during each DNA extraction. All genomic DNA was frozen at −80 °C until sequencing. Extracted DNA (10 ng) was prepared for 16S amplicon sequencing by the MiSeq platform using the Illumina protocol (Part # 15044223, Rev. B) with modifications to ensure sufficient amplification of low amounts of DNA. The V3-V4 region of the 16S ribosomal RNA gene was amplified using the primers forward (5′-CCTACGGGNGGCWGCAG-3′) and reverse (5′-GACTACHVGGGTATCTAATCC-3′) [119]. All PCR products were purified by Agencourt AMPure XP magnetic beads (Beckman Coulter). The samples and mock community aliquots were then barcoded by Illumina’s dual indexing strategy (Nextera XT Index Kit v2, Sets A and B, Illumina) by using the default barcode layout from the Illumina Experiment Manager software v1.13.1, as described in the Illumina protocol. The quality of PCR products was assessed by Agilent 2100 Bioanalyzer (Agilent Technologies, Palo Alto, CA, USA). The amplicon libraries underwent further purification and quality checking, followed by dilution and equimolar pooling. Finally, 12 pM of the library mixtures, spiked with 20% PhiX control, was paired-end (2 × 300) sequenced using the MiSeq platform (Illumina, San Diego, CA, USA) at the Centro Servizi - B.R.I.T. (University of Catania).

### 4.9. Processing and Analyses of Sequencing Data

QIIME pipeline (Quantitative Insights into Microbial Ecology) v.1.9.1 was used to process the generated raw FASTQ files [120]. V3-V4 16S rRNA FASTQ were de-multiplexed using the barcodes. The paired-end sequences were assembled to form a single read using FLASH [121] and quality-filtered ≥80% bases in a read above Q30 (see Supplementary Table S4). Merged reads were length-filtered based on 445 bp (the expected length). The ends of retained (not-merged) forward reads were clipped to a total read length of 270 bp to remove low quality bases. The high-quality reads were clustered against a reference sequence collection with QIIME. To focus only on the prominent taxa, a filtering step of 0.01% at the Operational taxonomic Unit (OTU) level was performed by running a workflow on QIIME (*filter_otus_from_otu_table.py*). The taxonomy of each 16S rRNA gene sequence was collapsed to OTUs using the open reference-based OTU picking method against Greengenes database at 97% of sequence similarity [122]. Chimeras were identified and removed by Chimera Slayer and the UCHIME algorithm. Any reads that did not match the reference sequence collection were subsequently clustered de novo. To avoid sample size biases in downstream analyses, rarefaction curves were generated with QIIME (*alpha_rarefaction.py* workflow) and calculated by applying Explicet and a maximum depth of 74.469 sequences/sample [123]. The OTU tables were used for assessing α-diversity indices (Chao-1, Shannon and Shannon evenness) calculated from the taxonomic profiles and compared across the ASD and NUC groups by QIIME algorithms. Independent Student’s *t*-test and Mann–Whitney U test were used to evaluate α-diversity among the taxonomic profiles and compared across the ASD and NUC groups. β-diversity between ASD and NUC groups was analyzed by weighted and unweighted UniFrac distance matrices (*beta_diversity.py* workflow) and visualized through tridimensional PCoA plot by using EMPeror (http://boocore.github.io/emperor/). The differentially abundant OTUs across two sample categories were identified by QIIME scripts (differential_abundance.py). The core microbiome was determined by QIIME algorithms (*compute_core_microbiome.py*) and the diversity analysis was performed with the script *core_diversity_analyses.py*.

DNA sequences were deposited in the Sequence Read Archive under BioProjects PRJNA518756 and PRJNA518760.

### 4.10. Correlation and Negative Binomial Regression Analyses

To investigate whether a linear relationship exists between the differential expression of salivary miRNAs, microbiome taxa and the neuropsychological and hematological parameters for each selected participant, correlation analysis was performed. Additionally, we applied a Negative Binomial (NB) regression to explore the potential effects of the clinical parameters and the miRNA expression (potential predictors) on the relative microbial abundances (outcome variables). In particular, relationships were evaluated by using generalized linear models (GLM) assuming a negative binomial distribution and log link function, since our response variable is represented by over-dispersed count data that didn’t have an excessive number of zeros [124].

### 4.11. Computational Enrichment Analysis

To investigate the functional meaning and potential ASD association of the identified DE miRNAs, DIANA-mirPath v.3 web server [125] and miRNet tool [126] were used for pathway enrichment analysis from KEGG (Kyoto Encyclopedia of Genes and Genomes) and Reactome gene annotation databases.

### 4.12. Statistical Approach

For miRNA profiling analysis, SAM (Significance of Microarrays Analysis) statistical tests were computed by using MeV (Multi experiment viewer v4.8.1) statistical analysis software. We computed a two-class unpaired test, based on 100 permutations. Fold change (FC) values were obtained by calculating the ratio between the normalized count mean of each group. MeV was also used to generate a heat-map of identified DE-miRNAs.

Concerning microbiome analyses, OTU frequencies across sample groups were performed by the Kruskal–Wallis test. Statistical analysis of taxonomic profiles was performed using STAMP (Software Testing AMPlification) [127,128,129] by a two-sided White’s non-parametric *t*-test. Extended error bar plots were produced by STAMP (White’s non-parametric *t*-test and *p*-value < 0.05) showing the bacterial taxa with a significant difference (*p*-value < 0.05). Mann–Whitney–Kruskal–Wallis, *t*-test/ANOVA were used for confirmation (Supplementary Table S1).

The correction for multiple tests of high throughput analyses was performed by applying Benjamini–Hochberg FDR (False Discovery Rate).

The Mann–Whitney U test was applied to evaluate the differential expression of tested miRNAs between the two groups in the validation analysis (*p*-value < 0.05). Cliff’s delta statistic [130,131] was used to estimate the non-parametric effect sizes and were calculated by using the Cliff’s Delta Calculator by Macbeth et al. [132].

Correlation analyses were performed by applying Spearman test with two-sided *p*-values corrected for multiple comparisons by using the Bonferroni-Šídák approach. Statistical analyses were computed using GraphPad Prism version 7.00 for Windows (GraphPad Software, La Jolla, CA, USA). To determine the strength of associations between variables, the correlation coefficients themselves were interpreted as index of effect size [133].

Negative binomial, multiple and simple linear regression analyses (*p* < 0.05) were performed by IBM SPSS Statistics 25 software. The effect sizes were provided by RcountD (https://stefany.shinyapps.io/RcountD). For functional enrichment analysis, Fisher’s exact *t*-test (*p* < 0.05) was used.

## Figures and Tables

**Table 1:** Demographic, neuropsychological, and hematological characteristics of the clinical samples.

	ASD	NUC
**Number of Participants**	76	39
Sex (M:F)	60:16	28:11
Age (years)	6.9 (±1.5)	6.9 (±1.8)
Time of collection (h)	09:58 (±00:28)	10:03 (±00:30)
Time since last meal (h)	2.99 (±0.12)	2.98 (±0.13)
**IQ**		
TIQ	69.6 (±19.3)	96.7 (±11.6)
VIQ	67.8 (±19.7)	96.2 (±13.3)
PIQ	71 (±21.4)	97.9 (±12.7)
ADOS		
A	4.6 (±1.9)	0
B	7.7 (±2.4)	0
C	2.1 (±1.2)	0
D	2.6 (±1.3)	0
ADI-R		
A	10.9 (±3.7)	0
B	9.4 (±2.5)	0
C	5.9 (±2.8)	0
D	3.2 (±1.3)	0
Prolactine	229.8 (±89.5)	/
Ceruloplasmin	27.6 (±5.6)	/
Lactate	15.3 (±4.8)	/
Ammonium	24.7 (±9.3)	/
TSH	2.3 (±0.9)	/

Data are shown as means and ± standard deviations between brackets. IQ: Intelligence Quotient; TIQ: Total Intelligence Quotient; VIQ: Verbal Intelligence Quotient; PIQ: Performance Intelligence Quotient; ADOS: Autism Diagnostic Observation Schedule; ADOS-A: Communication; ADOS-B: Social Interaction; ADOS-C: Imagination; ADOS-D: Repetitive and Restricted Behavior; ADI-R: Autism Diagnostic Interview-Revised; ADI-A: Qualitative anomalies in social interaction; ADI-B: Qualitative anomalies in communication; ADI-C: Repetitive and restricted behavior; ADI-D: Anomalies in neurodevelopment arisen before 36 months old; TSH: Thyroid stimulant hormone. According to two-tailed Mann–Whitney U test, there were no statistically significant differences between groups in sex (*p* = 0.3496), age (*p* = 0.6731), time of sample collection (*p* = 0.2008), and time since last meal (*p* = 0.4456).

**Table 2:** Validation analysis of miRNA expression in saliva.

DE miRNA ASD vs. NUC	FC	Mann–Whitney Test *p-Value*
let-7b-5p	−1.99	0.0002
miR-16-5p	−1.68	0.0002
miR-29a-3p	1.43	0.0123
miR-141-3p	2.93	0.0431
miR-451a	−3.58	<0.0001

## Supplementary Materials

The following are available online at https://www.mdpi.com/1422-0067/21/17/6203/s1.

## Abbreviations

ADI-R	Autism Diagnostic Interview—Revised
ADOS	Autism Diagnostic Observation Schedule
ASD	Autistic Spectrum Disorder
CNS	Central Nervous System
CNVs	Copy Number Variants
CSF	cerebrospinal fluid
df	Degree of freedom
DE	Differentially expressed
DSM-5	Diagnostic and Statistical Manual of Mental Disorders, 5th edition
FC	Fold Change
FDR	False Discovery Rate
FoxO	Forkhead box O
GLM	Generalized linear model
GMN	Global median normalization
IQ	Intelligence Quotient
KEGG	Kyoto Encyclopedia of Genes and Genomes
LR	Likelihood ratio
LDH	Lactate dehydrogenase
MeV	Multi experiment viewer
miRNA	microRNA
MLR	Multiple linear regression
NB	Negative binomial
NBR	Negative binomial regression
NUC	Neurologically unaffected control
NS	Non-significant
OTU	Operational taxonomic Unit
p	*p*-value
PCoA	Principal coordinate analysis
PIQ	Performance Intelligence Quotient
QIIME	Quantitative Insights Into Microbial Ecology
RQ	Relative quantification
rRNA	Ribosomal RNA
RT-PCR	Real Time-polymerase chain reactions
SAM	Significance of Microarrays Analysis
SLR	Simple linear regression
SMD	Standardized mean difference
TIQ	Total Intelligence Quotient
TLR-7	Tall-like Receptor 7
TSH	Thyroid-stimulating Hormone
VIQ	Verbal Intelligence Quotient
WISC-III	Wechsler Intelligence Scale for Children, III edition
WPSSI	Wechsler Preschool and Primary Scale of Intelligence
YOAD	Young-Onset Alzheimer Disease.
