# Supplementary material for: Potential Associations Among Alteration of Salivary miRNAs, Saliva Microbiome Structure, and Cognitive Impairments in Autistic Children
Source: Int J Mol Sci. 2020 Aug 27;21(17):6203. doi: 10.3390/ijms21176203 (PMC7504581; doi:10.3390/ijms21176203)
Supplement: Supplementary file 1 [file ijms-21-06203-s001.zip › Supplementary Table S2.pdf]

Potential associations among alteration of salivary miRNAs, saliva microbiome structure and cognitive impairments in autistic children

Marco Ragusa[1,2§], Maria Santagati[3§], Federica Mirabella[1§], Giovanni Lauretta[1], Matilde Cirnigliaro[1], Duilia Brex[1], Cristina Barbagallo[1], Carla Noemi Domini[4], Mariangela Gulisano[4], Rita Barone[4], Laura Trovato[3], Salvatore Oliveri[3], Gino Mongelli[3,5], Ambra Spitale[3], Davide Barbagallo[1], Cinzia Di Pietro[1†], Stefania Stefani[3†], Renata Rizzo[4†], Michele Purrello[1*].

**Species**	*Actinobacillus parahaemolyticus*				
**Model results**	**B**	**Lower 95% CL**	**Upper 95% CL**	**p-value**	**SMD effect size**
**let-7b-5p**	1.050	-0.673	2.774	0.232	1.387
**miR-451a**	-0.851	-1.866	0.165	0.101	-0.428
**miR-29a-3p**	-0.244	-0.824	0.335	0.409	-0.162
**miR-16-5p**	-0.16	-0.856	0.535	0.651	-0.110
**miR-141-3p**	0.059	0.028	0.09	**0.000**	0.045
**VIQ**	-0.02	-0.109	0.069	0.659	-0.015
**PIQ**	-0.09	-0.194	0.013	0.088	-0.064
**TIQ**	0.119	-0.063	0.301	0.200	0.094
**ADI-A**	0.278	-0.015	0.571	0.063	0.239
**ADI-B**	-0.049	-0.446	0.349	0.81	-0.036
**ADI-C**	-0.473	-0.706	-0.24	**0.000**	-0.281
**ADI-D**	0.498	0.12	0.877	**0.010**	0.482
**ADOS-A**	0.086	-0.297	0.469	0.66	0.067
**ADOS-B**	-0.023	-0.298	0.253	0.870	-0.017
**ADOS-C**	-0.602	-1.222	0.017	0.057	-0.338
**ADOS-D**	0.577	-0.093	1.247	0.091	0.583
**Ammonium**	-0.145	-0.216	-0.074	**0.000**	-0.101
**Lactate**	0.142	0.035	0.249	**0.009**	0.114
**Ceruloplasmin**	-0.121	-0.224	-0.019	**0.021**	-0.085
**Prolactin**	0.000	-0.005	0.005	0.904	0.000
**TSH**	0.262	-0.388	0.912	0.430	0.224

**Species**	*Filifactor spp*				
**Model results**	**B**	**Lower 95% CL**	**Upper 95% CL**	**p-value**	**SMD effect size**
**let-7b-5p**	-0.886	0.7233	-2.303	0.221	-0.429
**miR-451a**	-0.512	0.5175	-1.527	0.322	-0.293
**miR-29a-3p**	0.455	0.2417	-0.019	0.06	0.421
**miR-16-5p**	0.227	0.3344	-0.428	0.497	0.186
**miR-141-3p**	-0.061	0.0157	-0.092	**0.000**	-0.043
**VIQ**	-0.064	0.0472	-0.156	0.177	-0.045
**PIQ**	-0.098	0.0482	-0.193	**0.042**	-0.068
**TIQ**	0.123	0.082	-0.038	0.133	0.096
**ADI-A**	0.236	0.1223	-0.003	0.053	0.194
**ADI-B**	-0.072	0.2043	-0.473	0.723	-0.051
**ADI-C**	0.101	0.122	-0.139	0.410	0.078
**ADI-D**	-0.295	0.2066	-0.700	0.153	-0.187
**ADOS-A**	-0.605	0.2051	-1.007	**0.003**	-0.332
**ADOS-B**	-0.263	0.1434	-0.544	0.067	-0.169
**ADOS-C**	0.390	0.228	-0.057	0.087	0.349
**ADOS-D**	-0.215	0.219	-0.645	0.326	-0.141

**Ammonium**	0.002	0.037	-0.07	0.95	0.001
**Lactate**	-0.041	0.0559	-0.150	0.467	-0.029
**Ceruloplasmin**	0.068	0.0708	-0.071	0.336	0.051
**Prolactin**	0.003	0.0027	-0.002	0.296	0.002
**TSH**	-0.326	0.4127	-1.135	0.429	-0.203

**Species**	***Moryella spp***				
**Model results**	**B**	**Lower 95% CL**	**Upper 95% CL**	**p-value**	**SMD effect size**
**let-7b-5p**	-0.079	-0.875	0.717	0.846	-0.090
**miR-451a**	-0.450	-0.952	0.052	0.079	-0.439
**miR-29a-3p**	0.045	-0.22	0.311	0.739	0.056
**miR-16-5p**	0.064	-0.257	0.384	0.697	0.08
**miR-141-3p**	-0.051	-0.069	-0.033	**0.000**	-0.06
**VIQ**	-0.034	-0.079	0.011	0.141	-0.041
**PIQ**	-0.031	-0.077	0.015	0.185	-0.037
**TIQ**	0.055	-0.02	0.13	0.148	0.069
**ADI-A**	-0.128	-0.329	0.074	0.215	-0.146
**ADI-B**	-0.047	-0.293	0.199	0.71	-0.056
**ADI-C**	-0.051	-0.177	0.075	0.429	-0.06
**ADI-D**	-0.130	-0.367	0.106	0.281	0.148
**ADOS-A**	0.044	-0.128	0.216	0.616	0.055
**ADOS-B**	-0.158	-0.331	0.014	0.072	-0.177
**ADOS-C**	0.152	-0.158	0.462	0.335	0.199
**ADOS-D**	-0.098	-0.372	0.176	0.482	-0.113
**Ammonium**	0.013	-0.024	0.049	0.496	0.016
**Lactate**	0.020	-0.036	0.075	0.492	0.024
**Ceruloplasmin**	0.106	0.035	0.178	**0.003**	0.136
**Prolactin**	0.003	0.000	0.006	**0.022**	0.004
**TSH**	0.003	-0.391	0.397	0.988	0.004

**Species**	***Pasteurellaceae***				
**Model results**	**B**	**Lower 95% CL**	**Upper 95% CL**	**p-value**	**SMD effect size**
**let-7b-5p**	0.734	-0.599	2.066	0.281	0.939
**miR-451a**	-0.023	-0.686	0.639	0.945	-0.02
**miR-29a-3p**	0.159	-0.228	0.547	0.421	0.149
**miR-16-5p**	-0.272	-0.844	0.300	0.351	-0.206
**miR-141-3p**	0.021	-0.009	0.052	0.175	0.018
**VIQ**	0.020	-0.060	0.100	0.617	0.018
**PIQ**	0.082	-0.005	0.170	0.064	0.074
**TIQ**	-0.082	-0.233	0.068	0.285	-0.068
**ADI-A**	-0.117	-0.421	0.188	0.454	-0.096
**ADI-B**	-0.197	-0.584	0.191	0.32	-0.155
**ADI-C**	-0.134	-0.337	0.068	0.194	-0.109

**ADI-D**	0.406	0.008	0.804	**0.046**	0.434
**ADOS-A**	-0.258	-0.552	0.037	0.087	-0.197
**ADOS-B**	0.199	-0.049	0.448	0.116	0.191
**ADOS-C**	0.055	-0.406	0.517	0.814	0.049
**ADOS-D**	0.400	-0.099	0.899	0.116	0.426
**Ammonium**	-0.029	-0.089	0.030	0.334	-0.025
**Lactate**	0.082	-0.002	0.167	0.057	0.074
**Ceruloplasmin**	-0.100	-0.184	-0.017	**0.019**	-0.082
**Prolactin**	0.000	-0.005	0.004	0.866	0.000
**TSH**	-0.294	-0.884	0.296	0.329	-0.221

**Species**	***Ralstonia spp***				
**Model results**	**B**	**Lower 95% CL**	**Upper 95% CL**	**p-value**	**SMD effect size**
**let-7b-5p**	-1.763	-4.475	0.950	0.203	-0.284
**miR-451a**	-2.747	-6.132	0.637	0.112	-0.321
**miR-29a-3p**	0.419	-0.130	0.967	0.135	0.178
**miR-16-5p**	1174	-0.067	2.416	0.064	0.766
**miR-141-3p**	-0.049	-0.102	0.003	0.063	-0.016
**VIQ**	0.233	0.085	0.380	**0.002**	0.090
**PIQ**	0.151	0.014	0.288	**0.031**	0.056
**TIQ**	-0.350	-0.592	-0.107	**0.005**	-0.101
**ADI-A**	0.102	-0.424	0.627	0.704	0.037
**ADI-B**	0.107	-0.592	0.805	0.765	0.039
**ADI-C**	-0.024	-0.368	0.321	0.893	-0.008
**ADI-D**	-0.027	-0.748	0.694	0.942	-0.009
**ADOS-A**	-0.372	-1.045	0.301	0.278	-0.106
**ADOS-B**	0.120	-0.383	0.622	0.64	0.044
**ADOS-C**	0.095	-0.767	0.958	0.828	0.034
**ADOS-D**	0.38	-0.446	1.206	0.368	0.158
**Ammonium**	0.077	-0.028	0.183	0.152	0.027
**Lactate**	-0.036	-0.275	0.203	0.767	-0.012
**Ceruloplasmin**	0.119	-0.055	0.292	0.180	0.043
**Prolactin**	-0.009	-0.019	0.001	0.066	-0.003
**TSH**	-0.679	-1.985	0.627	0.308	-0.169

**Species**	***Tannerella spp***				
**Model results**	**B**	**Lower 95% CL**	**Upper 95% CL**	**p-value**	**SMD effect size**
**let-7b-5p**	-1.130	-1.868	-0.405	**0.002**	-0.875
**miR-451a**	-0.008	-0.603	0.588	0.980	-0.010
**miR-29a-3p**	-0.095	-0.360	0.170	0.482	-0.117
**miR-16-5p**	0.429	0.148	0.709	**0.003**	0.690
**miR-141-3p**	-0.035	-0.052	-0.018	**0.000**	-0.044
**VIQ**	-0.048	-0.093	-0.002	**0.040**	-0.060

**PIQ**	-0.059	-0.103	-0.015	**0.008**	-0.074
**TIQ**	0.089	0.011	0.167	**0.025**	0.12
**ADI-A**	0.069	-0.073	0.212	0.339	0.092
**ADI-B**	0.014	-0.205	0.232	0.903	0.018
**ADI-C**	0.146	0.016	0.275	**0.028**	0.203
**ADI-D**	-0.162	-0.379	0.056	0.145	-0.193
**ADOS-A**	0.181	0.005	0.357	**0.044**	0.256
**ADOS-B**	-0.195	-0.342	-0.049	**0.009**	-0.228
**ADOS-C**	0.146	-0.138	0.430	0.313	0.203
**ADOS-D**	0.017	-0.217	0.251	0.888	0.022
**Ammonium**	0.024	-0.011	0.059	0.183	0.031
**Lactate**	-0.001	-0.055	0.052	0.957	-0.001
**Ceruloplasmin**	0.024	-0.039	0.087	0.447	0.031
**Prolactin**	3.000	-0.003	0.003	0.955	-0.104
**TSH**	-0.073	-0.461	0.315	0.711	-0.091

**Species**	***Weeksellaceae***				
**Model results**	**B**	**Lower 95% CL**	**Upper 95% CL**	**p-value**	**SMD effect size**
**let-7b-5p**	0.123	-0.529	0.774	0.712	0.190
**miR-451a**	-0.580	-0.941	-0.219	**0.002**	-0.638
**miR-29a-3p**	0.06	-0.167	0.286	0.606	0.09
**miR-16-5p**	0.046	-0.218	0.309	0.734	0.068
**miR-141-3p**	0.026	0.011	0.040	**0.001**	0.038
**VIQ**	-0.061	-0.099	-0.022	**0.002**	-0.086
**PIQ**	-0.038	-0.077	0.000	**0.049**	-0.054
**TIQ**	0.086	0.02	0.153	**0.0110**	0.13
**ADI-A**	0.071	-0.053	0.195	0.263	0.107
**ADI-B**	-0.183	-0.374	0.009	0.062	-0.242
**ADI-C**	0.050	-0.050	0.149	0.326	0.074
**ADI-D**	-0.070	-0.274	0.134	0.501	-0.098
**ADOS-A**	-0.008	-0.163	0.148	0.924	-0.012
**ADOS-B**	0.016	-0.115	0.146	0.813	0.023
**ADOS-C**	-0.196	-0.394	0.002	0.052	-0.051
**ADOS-D**	-0.036	-0.268	0.196	0.762	-0.051
**Ammonium**	-0.028	-0.058	0.001	0.061	-0.04
**Lactate**	0.004	-0.041	0.049	0.862	0.006
**Ceruloplasmin**	-0.003	-0.050	0.045	0.918	-0.004
**Prolactin**	0.001	-0.002	0.003	0.557	0.001
**TSH**	0.063	-0.261	0.387	0.702	0.094

Supplementary Table S2. Parameter estimates from the negative binomial regression model predicting abundances of microbiome species: regression coefficient (B); lower and upper limits of

the 95% Wald confidence interval; p-values calculated by Wald Chi-Square test; Standardized mean difference (SMD) effect size. Statistically significant p-values are indicated in bold.
