# Supplementary material for: Potential Associations Among Alteration of Salivary miRNAs, Saliva Microbiome Structure, and Cognitive Impairments in Autistic Children
Source: Int J Mol Sci. 2020 Aug 27;21(17):6203. doi: 10.3390/ijms21176203 (PMC7504581; doi:10.3390/ijms21176203)
Supplement: Supplementary file 1 [file ijms-21-06203-s001.zip › Supplementary Table S3.pdf]

Potential associations among alteration of salivary miRNAs, saliva microbiome structure and cognitive impairments in autistic children

Marco Ragusa[1,2§], Maria Santagati[3§], Federica Mirabella[1§], Giovanni Lauretta[1], Matilde Cirnigliaro[1], Duilia Brex[1], Cristina Barbagallo[1], Carla Noemi Domini[4], Mariangela Gulisano[4], Rita Barone[4], Laura Trovato[3], Salvatore Oliveri[3], Gino Mongelli[3,5], Ambra Spitale[3], Davide Barbagallo[1], Cinzia Di Pietro[1†], Stefania Stefani[3†], Renata Rizzo[4†], Michele Purrello[1*].

**Species**	***Actinobacillus parahaemolyticus***					
**Type of model**	**NBR**		**MLR**		**SLR**	
**Model results**	**B**	**p-value**	**B**	**p-value**	**B**	**p-value**
**let-7b-5p**	1.050	0.232	1.094	**0.006**	-0.007	0.726
**miR-451a**	-0.851	0.101	-0.217	0.323	-0.025	0.355
**miR-29a-3p**	-0.244	0.409	-0.178	0.151	-0.016	0.656
**miR-16-5p**	-0.160	0.651	-0.318	**0.041**	-0.003	0.696
**miR-141-3p**	0.059	**0.000**	0.030	**0.001**	0.027	**0.000**
**VIQ**	-0.020	0.659	0.002	0.927	-0.005	0.270
**PIQ**	-0.090	0.088	-0.046	**0.043**	-0.006	0.186
**TIQ**	0.119	0.200	0.048	0.201	-0.004	0.421
**ADI-A**	0.278	0.063	-0.010	0.891	0.032	0.110
**ADI-B**	-0.049	0.810	0.192	0.108	0.042	0.070
**ADI-C**	-0.473	**0.000**	-0.174	**0.006**	0.021	0.472
**ADI-D**	0.498	**0.010**	0.103	0.379	0.117	**0.032**
**ADOS-A**	0.086	0.660	0.006	0.949	0.065	0.118
**ADOS-B**	-0.023	0.870	0.014	0.859	0.049	**0.043**
**ADOS-C**	-0.602	0.057	-0.191	0.152	0.093	0.185
**ADOS-D**	0.577	0.091	0.163	0.211	0.118	0.052
**Ammonium**	-0.145	**0.000**	-0.035	**0.044**	0.002	0.901
**Lactate**	0.142	**0.009**	0.034	0.253	0.033	0.286
**Ceruloplasmin**	-0.121	**0.021**	-0.023	0.427	-0.006	0.827
**Prolactin**	0.000	0.904	0.000	0.900	0.001	0.535
**TSH**	0.262	0.430	0.333	0.082	0.063	0.726

**Species**	***Filifactor spp***					
**Type of model**	**NBR**		**MLR**		**SLR**	
**Model results**	**B**	**p-value**	**B**	**p-value**	**B**	**p-value**
**let-7b-5p**	-0.886	0.221	*-0.045*	*0.423*	-0.002	0.384
**miR-451a**	-0.512	0.322	*0.035*	*0.281*	-0.002	0.543
**miR-29a-3p**	0.455	0.060	*0.039*	***0.040***	0.002	0.664
**miR-16-5p**	0.227	0.497	*0.000*	*0.997*	-0.001	0.622
**miR-141-3p**	-0.061	**0.000**	*-0.003*	***0.035***	-0.001	0.335
**VIQ**	-0.064	0.177	*-0.004*	*0.255*	-0.001	**0.049**
**PIQ**	-0.098	**0.042**	*-0.008*	***0.014***	-0.002	**0.001**
**TIQ**	0.123	0.133	*0.010*	*0.068*	-0.002	**0.004**
**ADI-A**	0.236	0.053	*0.022*	*0.055*	0.007	**0.007**
**ADI-B**	-0.072	0.723	*-0.022*	*0.211*	0.007	**0.034**
**ADI-C**	0.101	0.410	*-0.005*	*0.611*	0.008	**0.040**
**ADI-D**	-0.295	0.153	*-0.009*	*0.623*	0.015	**0.044**
**ADOS-A**	-0.605	**0.003**	*-0.015*	*0.297*	0.006	0.269
**ADOS-B**	-0.263	0.067	*-0.020*	*0.103*	0.005	0.141
**ADOS-C**	0.390	0.087	*0.003*	*0.862*	0.013	0.177
**ADOS-D**	-0.215	0.326	*0.008*	*0.671*	0.015	0.059

**Ammonium**	0.002	0.950	*-0.001*	*0.572*	-0.003	0.190
**Lactate**	-0.041	0.467	*-0.005*	*0.274*	-0.007	0.060
**Ceruloplasmin**	0.068	0.336	*0.001*	*0.869*	-0.002	0.510
**Prolactin**	0.003	0.296	*0.000*	*0.373*	0.000	0.763
**TSH**	-0.326	0.429	*0.006*	*0.841*	0.030	0.196

**Species**	***Moryella spp***					
**Type of model**	**NBR**		**MLR**		**SLR**	
**Model results**	**B**	**p-value**	**B**	**p-value**	**B**	**p-value**
**let-7b-5p**	-0.079	0.846	*-0.063*	*0.623*	-0.001	0.845
**miR-451a**	-0.450	0.079	*0.009*	*0.900*	-0.006	0.528
**miR-29a-3p**	0.045	0.739	*-0.016*	*0.695*	-0.019	0.141
**miR-16-5p**	0.064	0.697	*0.020*	*0.705*	-0.002	0.418
**miR-141-3p**	-0.051	**0.000**	*-0.006*	***0.049***	-0.005	**0.029**
**VIQ**	-0.034	0.141	*-0.006*	*0.398*	0.005	**0.001**
**PIQ**	-0.031	0.185	*-0.003*	*0.738*	0.005	**0.002**
**TIQ**	0.055	0.148	*0.009*	*0.465*	0.005	**0.001**
**ADI-A**	-0.128	0.215	*-0.019*	*0.458*	-0.018	**0.010**
**ADI-B**	-0.047	0.710	*0.021*	*0.599*	-0.019	**0.020**
**ADI-C**	-0.051	0.429	*0.006*	*0.786*	-0.023	**0.027**
**ADI-D**	-0.130	0.281	*-0.073*	*0.077*	-0.059	**0.002**
**ADOS-A**	0.044	0.616	*0.031*	*0.354*	-0.025	0.093
**ADOS-B**	-0.158	0.072	*-0.009*	*0.741*	-0.018	**0.041**
**ADOS-C**	0.152	0.335	*0.032*	*0.480*	-0.038	0.132
**ADOS-D**	-0.098	0.482	*-0.017*	*0.697*	-0.038	0.083
**Ammonium**	0.013	0.496	*0.009*	*0.134*	0.002	0.650
**Lactate**	0.020	0.492	*-0.013*	*0.196*	-0.011	0.174
**Ceruloplasmin**	0.106	**0.003**	*0.021*	***0.039***	0.017	**0.026**
**Prolactin**	0.003	**0.022**	*0.000*	*0.752*	0.000	0.901
**TSH**	0.003	0.988	*0.022*	*0.730*	-0.008	0.868

**Species**	***Pasteurellaceae***					
**Type of model**	**NBR**		**MLR**		**SLR**	
**Model results**	**B**	**p-value**	**B**	**p-value**	**B**	**p-value**
**let-7b-5p**	0.734	0.281	*0.069*	*0.664*	0.000	0.976
**miR-451a**	-0.023	0.945	*0.036*	*0.694*	-0.003	0.699
**miR-29a-3p**	0.159	0.421	*0.046*	*0.380*	0.009	0.461
**miR-16-5p**	-0.272	0.351	*-0.040*	*0.535*	0.000	0.873
**miR-141-3p**	0.021	0.175	*0.004*	*0.264*	0.005	**0.015**
**VIQ**	0.020	0.617	*0.003*	*0.737*	-0.002	0.077
**PIQ**	0.082	0.064	*0.006*	*0.508*	-0.002	0.154
**TIQ**	-0.082	0.285	*-0.006*	*0.692*	-0.002	0.137
**ADI-A**	-0.117	0.454	*-0.019*	*0.557*	0.006	0.360
**ADI-B**	-0.197	0.320	*-0.032*	*0.516*	0.010	0.183

**ADI-C**	-0.134	0.194	*-0.006*	*0.803*	0.009	0.354
**ADI-D**	0.406	**0.046**	*0.037*	*0.456*	0.030	0.085
**ADOS-A**	-0.258	0.087	*-0.027*	*0.509*	0.020	0.118
**ADOS-B**	0.199	0.116	*0.024*	*0.481*	0.016	**0.031**
**ADOS-C**	0.055	0.814	*0.044*	*0.428*	0.059	**0.006**
**ADOS-D**	0.400	0.116	*0.070*	*0.207*	0.045	**0.017**
**Ammonium**	-0.029	0.334	*-0.002*	*0.810*	0.002	0.783
**Lactate**	0.082	0.057	*0.006*	*0.615*	0.012	0.208
**Ceruloplasmin**	-0.100	**0.019**	*-0.026*	***0.040***	-0.011	0.212
**Prolactin**	0.000	0.866	*0.000*	*0.780*	0.000	0.908
**TSH**	-0.294	0.329	*-0.051*	*0.520*	-0.016	0.773

**Species**	***Ralstonia spp***					
**Type of model**	**NBR**		**MLR**		**SLR**	
**Model results**	**B**	**p-value**	**B**	**p-value**	**B**	**p-value**
**let-7b-5p**	-1.763	0.203	*-0.168*	*0.547*	0.007	0.513
**miR-451a**	-2.747	0.112	*-0.065*	*0.692*	0.003	0.831
**miR-29a-3p**	0.419	0.135	*0.062*	*0.500*	0.023	0.221
**miR-16-5p**	1.174	0.064	*0.066*	*0.558*	0.004	0.358
**miR-141-3p**	-0.049	0.063	*-0.003*	*0.575*	-0.002	0.501
**VIQ**	0.233	**0.002**	*0.001*	*0.965*	-0.003	0.211
**PIQ**	0.151	**0.031**	*-0.001*	*0.948*	-0.004	0.095
**TIQ**	-0.350	**0.005**	*-0.005*	*0.865*	-0.004	0.085
**ADI-A**	0.102	0.704	*0.019*	*0.737*	0.014	0.164
**ADI-B**	0.107	0.765	*-0.049*	*0.580*	0.014	0.230
**ADI-C**	-0.024	0.893	*0.017*	*0.702*	0.012	0.414
**ADI-D**	-0.027	0.942	*-0.006*	*0.941*	0.036	0.199
**ADOS-A**	-0.372	0.278	*0.058*	*0.419*	0.040	0.053
**ADOS-B**	0.120	0.640	*-0.051*	*0.392*	0.015	0.211
**ADOS-C**	0.095	0.828	*-0.017*	*0.862*	0.055	0.118
**ADOS-D**	0.380	0.368	*0.066*	*0.496*	0.045	0.143
**Ammonium**	0.077	0.152	*0.005*	*0.692*	-0.007	0.445
**Lactate**	-0.036	0.767	*-0.010*	*0.656*	-0.024	0.117
**Ceruloplasmin**	0.119	0.180	*0.036*	*0.098*	0.025	0.075
**Prolactin**	-0.009	0.066	*0.000*	*0.928*	0.000	0.764
**TSH**	-0.679	0.308	*-0.077*	*0.582*	-0.105	0.245

**Species**	***Tannerella spp***					
**Type of model**	**NBR**		**MLR**		**SLR**	
**Model results**	**B**	**p-value**	**B**	**p-value**	**B**	**p-value**
**let-7b-5p**	-1.130	**0.002**	*-0.076*	*0.215*	0.005	0.344
**miR-451a**	-0.008	0.980	*0.027*	*0.442*	0.003	0.652
**miR-29a-3p**	-0.095	0.482	*-0.010*	*0.632*	0.001	0.931
**miR-16-5p**	0.429	**0.003**	*0.024*	*0.326*	0.001	0.490

**miR-141-3p**	-0.035	**0.000**	*-0.003*	*0.055*	-0.003	0.083
**VIQ**	-0.048	**0.040**	*-0.006*	*0.067*	0.002	0.093
**PIQ**	-0.059	**0.008**	*-0.008*	***0.025***	0.002	0.064
**TIQ**	0.089	**0.025**	*0.013*	***0.040***	0.002	**0.049**
**ADI-A**	0.069	0.339	*0.017*	*0.167*	-0.010	**0.038**
**ADI-B**	0.014	0.903	*0.003*	*0.896*	-0.015	**0.011**
**ADI-C**	0.146	**0.028**	*0.009*	*0.382*	-0.013	0.080
**ADI-D**	-0.162	0.145	*-0.018*	*0.339*	-0.029	**0.036**
**ADOS-A**	0.181	**0.044**	*0.009*	*0.543*	-0.025	**0.015**
**ADOS-B**	-0.195	**0.009**	*-0.027*	***0.047***	-0.017	**0.006**
**ADOS-C**	0.146	0.313	*0.004*	*0.859*	-0.037	**0.031**
**ADOS-D**	0.017	0.888	*0.004*	*0.858*	-0.034	**0.025**
**Ammonium**	0.024	0.183	*0.002*	*0.439*	0.000	0.868
**Lactate**	-0.001	0.957	*-0.001*	*0.890*	-0.005	0.223
**Ceruloplasmin**	0.024	0.447	*0.005*	*0.302*	-0.001	0.731
**Prolactin**	-0.084	0.955	*0.000*	*0.981*	0.000	0.600
**TSH**	-0.073	0.711	*-0.004*	*0.883*	0.017	0.453

**Species**	***Weeksellaceae***					
**Type of model**	**NBR**		**MLR**		**SLR**	
**Model results**	**B**	**p-value**	**B**	**p-value**	**B**	**p-value**
**let-7b-5p**	0.123	0.712	0.325	0.192	-0.006	0.653
**miR-451a**	-0.580	**0.002**	-0.280	0.058	-0.013	0.468
**miR-29a-3p**	0.060	0.606	0.036	0.657	0.003	0.914
**miR-16-5p**	0.046	0.734	-0.071	0.474	-0.002	0.703
**miR-141-3p**	0.026	**0.001**	0.025	**0.000**	0.022	**0.000**
**VIQ**	-0.061	**0.002**	-0.026	0.059	-0.004	0.131
**PIQ**	-0.038	**0.049**	-0.025	0.089	-0.002	0.521
**TIQ**	0.086	**0.011**	0.047	0.058	-0.002	0.528
**ADI-A**	0.071	0.263	0.038	0.443	0.018	0.160
**ADI-B**	-0.183	0.062	-0.027	0.729	0.024	0.120
**ADI-C**	0.050	0.326	0.027	0.500	0.026	0.187
**ADI-D**	-0.070	0.501	-0.104	0.180	0.031	0.393
**ADOS-A**	-0.008	0.924	0.009	0.891	0.035	0.205
**ADOS-B**	0.016	0.813	0.000	0.999	0.020	0.219
**ADOS-C**	-0.196	0.052	-0.169	0.057	0.000	0.994
**ADOS-D**	-0.036	0.762	0.003	0.968	0.023	0.576
**Ammonium**	-0.028	0.061	-0.015	0.168	0.003	0.815
**Lactate**	0.004	0.862	0.008	0.667	0.021	0.287
**Ceruloplasmin**	-0.003	0.918	0.005	0.772	-0.001	0.940
**Prolactin**	0.001	0.557	0.000	0.624	0.000	0.954
**TSH**	0.063	0.702	0.030	0.806	-0.043	0.712

Supplementary Table S3. Comparison between parameters estimated from the negative binomial regression (NBR), multiple linear regression (MLR) and simple linear regression (SLR) models predicting abundances of microbiome species: regression coefficient (B); statistical significance of the independent variables (p-values). Data for those MLR which statistical significance of overall model was not reached ($p > 0.05$) are shown in italic. Statistically significant p-values are indicated in bold ($p < 0.05$).
