# Supplementary material for: Potential Associations Among Alteration of Salivary miRNAs, Saliva Microbiome Structure, and Cognitive Impairments in Autistic Children
Source: Int J Mol Sci. 2020 Aug 27;21(17):6203. doi: 10.3390/ijms21176203 (PMC7504581; doi:10.3390/ijms21176203)
Supplement: Supplementary file 1 [file ijms-21-06203-s001.zip › Supplementary Table S4.pdf]

Potential associations among alteration of salivary miRNAs, saliva microbiome structure and cognitive impairments in autistic children

Marco Ragusa[1,2§], Maria Santagati[3§], Federica Mirabella[1§], Giovanni Lauretta[1], Matilde Cirnigliaro[1], Duilia Brex[1], Cristina Barbagallo[1], Carla Noemi Domini[4], Mariangela Gulisano[4], Rita Barone[4], Laura Trovato[3], Salvatore Oliveri[3], Gino Mongelli[3,5], Ambra Spitale[3], Davide Barbagallo[1], Cinzia Di Pietro[1†], Stefania Stefani[3†], Renata Rizzo[4†], Michele Purrello[1*].

No	Sample	Read Pairs	Yield (Kbp)*	%Q30**	Mean Q†
1	ASD003	148,566	84,534	87.77	35.14
2	ASD005	130,436	74,218	87.94	35.16
3	ASD011	144,814	82,399	87.01	34.97
4	ASD014	125,809	71,585	87.02	34.96
5	ASD016	137,756	78,383	86.81	34.92
6	ASD020	124,683	70,944	87.93	35.17
7	ASD021	146,143	83,155	88.11	35.22
8	ASD023	108,866	61,944	88.40	35.29
9	ASD027	99,82	56,797	88.52	35.30
10	ASD028	99,622	56,684	87.50	35.08
11	ASD029	96,647	54,992	87.12	34.99
12	ASD030	97,502	55,478	87.82	35.15
13	ASD031	104,632	59,535	87.58	35.08
14	ASD032	87,086	49,551	87.58	35.09
15	ASD033	108,507	61,74	83.82	34.22
16	ASD034	108,913	61,971	88.17	35.23
17	ASD035	108,323	61,635	88.18	35.22
18	ASD037	167,607	95,368	87.49	35.07
19	ASD039	123,64	70,351	87.54	35.07
20	ASD041	117,894	67,081	87.03	34.97
21	ASD042	115,575	65,762	86.91	34.94
22	ASD043	120,787	68,727	86.92	34.95
23	ASD047	110,398	62,816	88.17	35.23
24	ASD048	125,261	71,273	87.60	35.10
25	ASD049	109,87	62,516	87.85	35.16
26	ASD050	169,879	96,661	87.61	35.10
27	ASD051	116,122	66,073	87.41	35.06
28	ASD052	91,188	51,885	87.59	35.11
29	ASD053	115,546	65,745	87.38	35.05
30	ASD054	118,956	67,685	88.43	35.29
31	ASD055	105,393	59,968	87.81	35.14
32	ASD056	99,169	56,427	88.40	35.28
33	ASD057	93,034	52,936	88.29	35.25
34	ASD058	103,7	59,005	87.49	35.08
35	ASD059	93,37	53,127	87.24	35.01
36	ASD060	91,262	51,928	86.53	34.86
37	ASD061	90,346	51,406	87.92	35.15
38	ASD062	90,172	51,307	87.98	35.18
39	ASD063	103,342	58,801	88.00	35.19
40	ASD064	139,269	79,244	87.71	35.13
41	ASD065	150,987	85,911	87.43	35.06
42	ASD066	145,56	82,823	87.24	35.02
43	ASD067	136,286	77,546	86.62	34.88
44	ASD068	145,483	82,779	87.64	35.10
45	ASD069	144,006	81,939	87.20	35.01
46	ASD070	145,072	82,545	87.54	35.09
47	ASD071	131,541	74,846	87.57	35.10
48	ASD072	169,413	96,395	87.81	35.15
49	ASD073	180,706	102,821	88.21	35.24
50	ASD074	151,876	86,417	87.32	35.04
51	ASD075	126,173	71,792	87.00	34.96
52	ASD076	120,554	68,595	86.88	34.95
53	ASD077	143,931	81,896	87.77	35.13
54	NUC001	149,7	85,179	87.96	35.18
55	NUC002	131,637	74,901	88.69	35.35
56	NUC003	100,436	57,148	86.95	34.96
57	NUC005	151,361	86,124	87.56	35.09

58	NUC007	148,574	84,538	87.24	35.01
59	NUC012	1,933,900	1,100,389	87.28	35.02
60	NUC013	158,13	89,975	86.73	34.89
61	NUC016	167,996	95,589	87.53	35.07
62	NUC017	150,135	85,426	87.95	35.17
63	NUC018	144,939	82,47	87.91	35.16
64	NUC023	140,838	80,136	86.99	34.96
65	NUC024	158,094	89,955	87.25	35.00
66	NUC025	143,326	81,552	87.01	34.96
67	NUC026	139,716	79,498	86.76	34.89
68	NUC027	131,401	74,767	85.88	34.70
69	NUC028	133,615	76,026	87.08	34.96
70	NUC029	133,202	75,791	87.37	35.05
71	NUC030	134,331	76,434	87.28	35.03
72	NUC031	152,8	86,943	87.91	35.17
73	NUC032	131,665	74,917	87.46	35.07
74	NUC033	150,454	85,608	87.62	35.11
75	NUC034	132,362	75,313	87.67	35.11
76	NUC035	124,624	70,911	87.52	35.08
77	NUC036	137,494	78,234	87.51	35.07
78	NUC037	129,168	73,496	87.91	35.17
79	NUC038	122,264	69,568	87.84	35.16
80	NUC040	142,348	80,996	88.10	35.21
**Total**		**10.919.413**	**1.330.145**	**87,50**	**35,07**

Supplementary Table S4. Various statistics describing the sorted reads of 80 saliva samples (Autism Spectrum Disorder, ASD n=53 and neurologically unaffected control, NUC =27).

All reads are passed filter, i.e. reads have passed the default Illumina filter procedure (chastity filter); *Yield (Kbp): number of bases called in kilobases. ** %Q30: percentage of bases with a quality score of at least 30 (inferred base call accuracy of 99.9%). † Mean Q: Q-score is a prediction of the probability of a wrong base call.
