# Supplementary material for: Potential Associations Among Alteration of Salivary miRNAs, Saliva Microbiome Structure, and Cognitive Impairments in Autistic Children
Source: Int J Mol Sci. 2020 Aug 27;21(17):6203. doi: 10.3390/ijms21176203 (PMC7504581; doi:10.3390/ijms21176203)
Supplement: Supplementary file 1 [file ijms-21-06203-s001.zip › Supplementary Figure S1.pdf]

Potential associations among alteration of salivary miRNAs, saliva microbiome structure and cognitive impairments in autistic children

Marco Ragusa[1,2§], Maria Santagati[3§], Federica Mirabella[1§], Giovanni Lauretta[1], Matilde Cirnigliaro[1], Duilia Brex[1], Cristina Barbagallo[1], Carla Noemi Domini[4], Mariangela Gulisano[4], Rita Barone[4], Laura Trovato[3], Salvatore Oliveri[3], Gino Mongelli[3,5], Ambra Spitale[3], Davide Barbagallo[1], Cinzia Di Pietro[1†], Stefania Stefani[3†], Renata Rizzo[4†], Michele Purrello[1*].

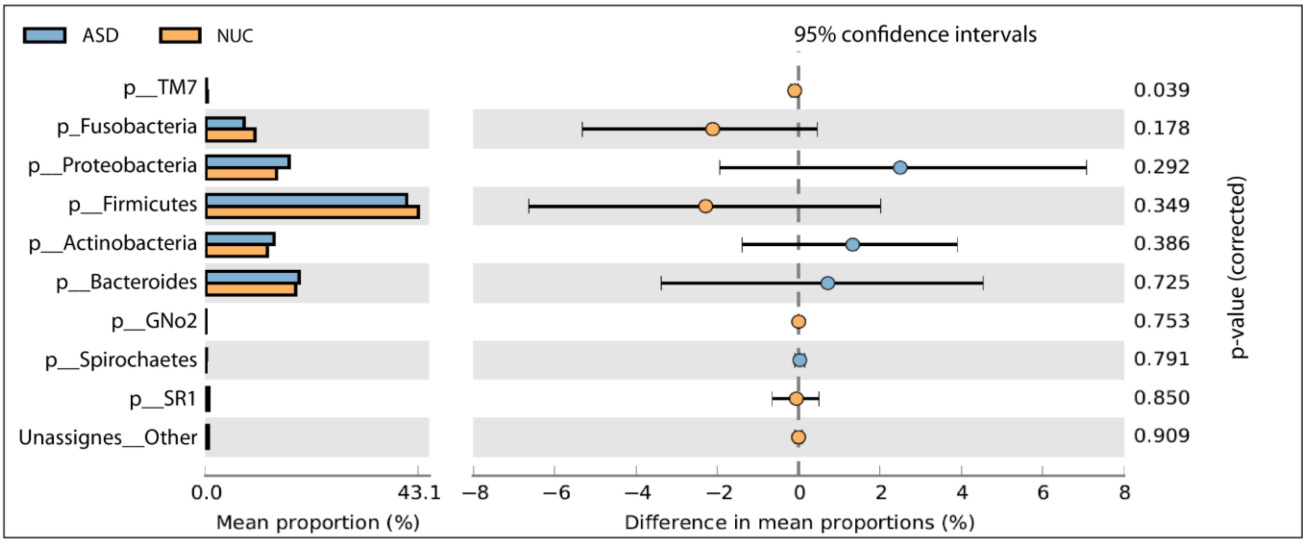

Supplementary Figure S1. The bacterial taxonomic abundance of all phyla in ASD and NC samples; this figure was produced using STAMP.
