# Supplementary material for: Potential Associations Among Alteration of Salivary miRNAs, Saliva Microbiome Structure, and Cognitive Impairments in Autistic Children
Source: Int J Mol Sci. 2020 Aug 27;21(17):6203. doi: 10.3390/ijms21176203 (PMC7504581; doi:10.3390/ijms21176203)
Supplement: Supplementary file 1 [file ijms-21-06203-s001.zip › Supplementary Figure S2.pdf]

Potential associations among alteration of salivary miRNAs, saliva microbiome structure and cognitive impairments in autistic children

Marco Ragusa[1,2§], Maria Santagati[3§], Federica Mirabella[1§], Giovanni Lauretta[1], Matilde Cirnigliaro[1], Duilia Brex[1], Cristina Barbagallo[1], Carla Noemi Domini[4], Mariangela Gulisano[4], Rita Barone[4], Laura Trovato[3], Salvatore Oliveri[3], Gino Mongelli[3,5], Ambra Spitale[3], Davide Barbagallo[1], Cinzia Di Pietro[1†], Stefania Stefani[3†], Renata Rizzo[4†], Michele Purrello[1*].

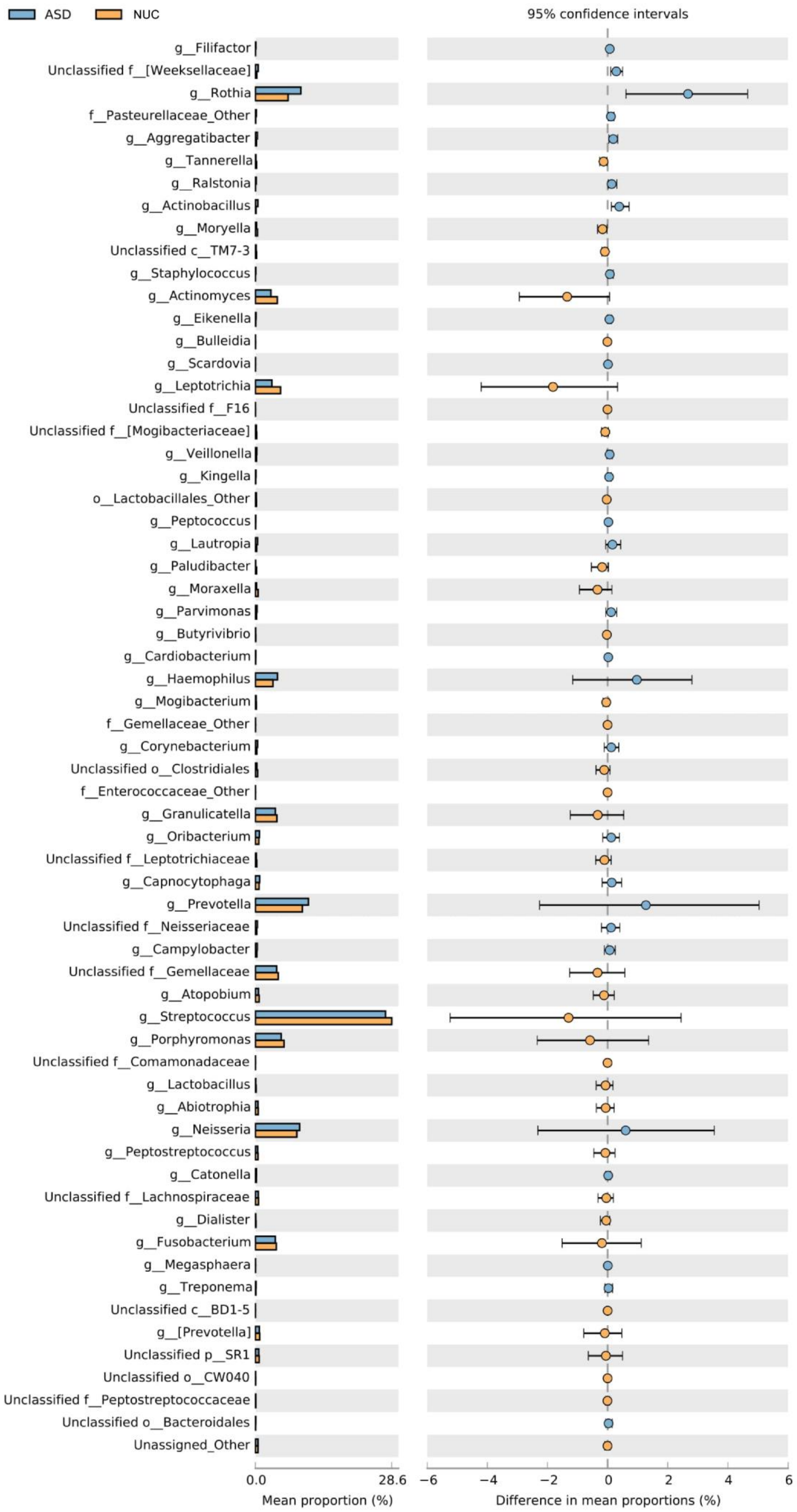

ASD
NUC
95% confidence intervals
g__Filifactor
Unclassified f__[Weeksellaceae]
g__Rothia
f__Pasteurellaceae_Other
g__Aggregatibacter
g__Tannerella
g__Ralstonia
g__Actinobacillus
g__Moryella
Unclassified c__TM7-3
g__Staphylococcus
g__Actinomyces
g__Eikenella
g__Bulleidia
g__Scardovia
g__Leptotrichia
Unclassified f__F16
Unclassified f__[Mogibacteriaceae]
g__Veillonella
g__Kingella
o__Lactobacillales_Other
g__Peptococcus
g__Lautropia
g__Paludibacter
g__Moraxella
g__Parvimonas
g__Butyrivibrio
g__Cardiobacterium
g__Haemophilus
g__Mogibacterium
f__Gemellaceae_Other
g__Corynebacterium
Unclassified o__Clostridiales
f__Enterococcaceae_Other
g__Granulicatella
g__Oribacterium
Unclassified f__Leptotrichiaceae
g__Capnocytophaga
g__Prevotella
Unclassified f__Neisseriaceae
g__Campylobacter
Unclassified f__Gemellaceae
g__Atopobium
g__Streptococcus
g__Porphyromonas
Unclassified f__Comamonadaceae
g__Lactobacillus
g__Abiotrophia
g__Neisseria
g__Peptostreptococcus
g__Catonella
Unclassified f__Lachnospiraceae
g__Dialister
g__Fusobacterium
g__Megasphaera
g__Treponema
Unclassified c__BD1-5
g__[Prevotella]
Unclassified p__SR1
Unclassified o__CW040
Unclassified f__Peptostreptococcaceae
Unclassified o__Bacteroidales
Unassigned_Other
0.0
28.6
Mean proportion (%)
−6
−4
−2
0
2
4
6
Difference in mean proportions (%)

Supplementary Figure S2. The bacterial taxonomic abundance of all genera in ASD and NUC samples; this figure was produced using STAMP.
