# Supplementary material for: Potential Associations Among Alteration of Salivary miRNAs, Saliva Microbiome Structure, and Cognitive Impairments in Autistic Children
Source: Int J Mol Sci. 2020 Aug 27;21(17):6203. doi: 10.3390/ijms21176203 (PMC7504581; doi:10.3390/ijms21176203)
Supplement: Supplementary file 1 [file ijms-21-06203-s001.zip › Supplementary Figure S3.pdf]

Potential associations among alteration of salivary miRNAs, saliva microbiome structure and cognitive impairments in autistic children

Marco Ragusa[1,2§], Maria Santagati[3§], Federica Mirabella[1§], Giovanni Lauretta[1], Matilde Cirnigliaro[1], Duilia Brex[1], Cristina Barbagallo[1], Carla Noemi Domini[4], Mariangela Gulisano[4], Rita Barone[4], Laura Trovato[3], Salvatore Oliveri[3], Gino Mongelli[3,5], Ambra Spitale[3], Davide Barbagallo[1], Cinzia Di Pietro[1†], Stefania Stefani[3†], Renata Rizzo[4†], Michele Purrello[1*].

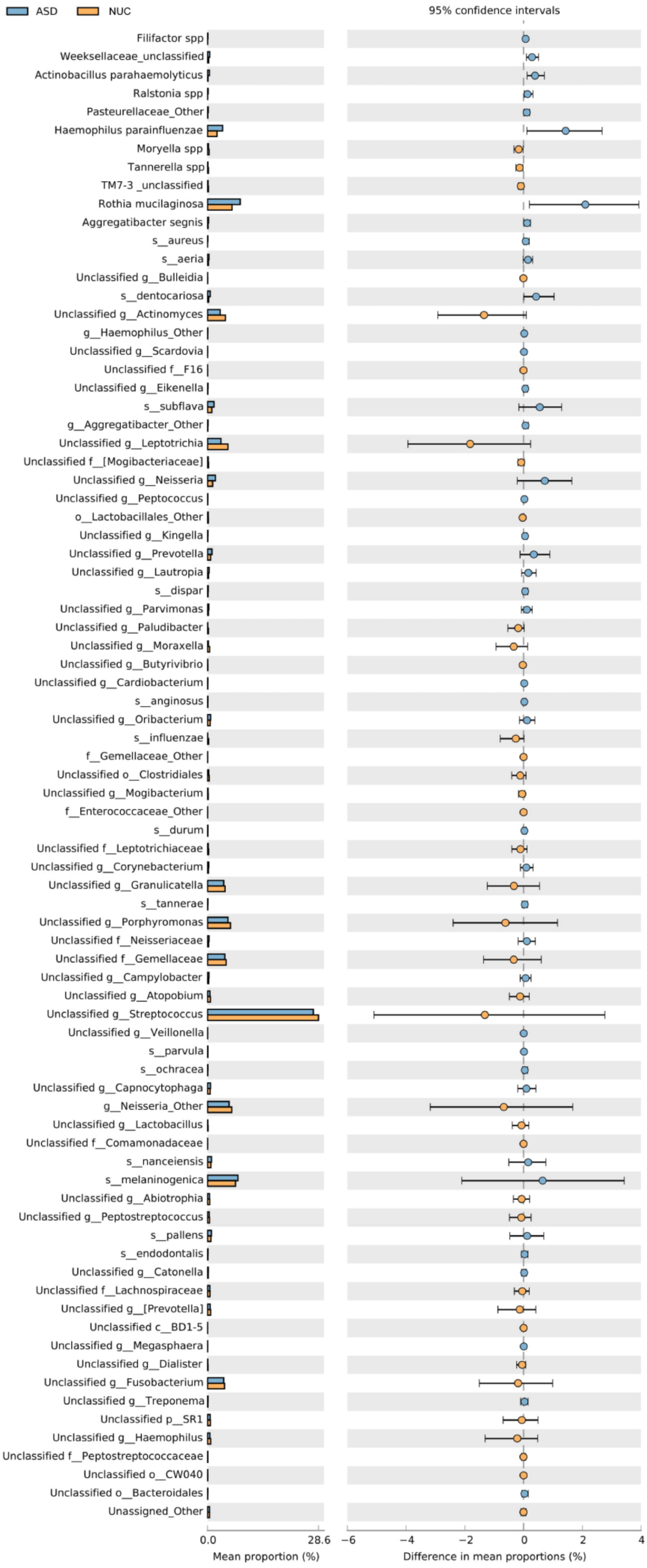
ASD
NUC
95% confidence intervals
Filifactor spp
Weeksellaceae_unclassified
Actinobacillus parahaemolyticus
Ralstonia spp
Pasteurellaceae_Other
Haemophilus parainfluenzae
Moryella spp
Tannerella spp
TM7-3 _unclassified
Rothia mucilaginosa
Aggregatibacter segnis
s__aureus
s__aeria
Unclassified g__Bulleidia
s__dentocariosa
Unclassified g__Actinomyces
g__Haemophilus_Other
Unclassified g__Scardovia
Unclassified f__F16
Unclassified g__Eikenella
s__subflava
g__Aggregatibacter_Other
Unclassified g__Leptotrichia
Unclassified f__[Mogibacteriaceae]
Unclassified g__Neisseria
Unclassified g__Peptococcus
o__Lactobacillales_Other
Unclassified g__Kingella
Unclassified g__Prevotella
Unclassified g__Lautropia
s__dispar
Unclassified g__Parvimonas
Unclassified g__Paludibacter
Unclassified g__Moraxella
Unclassified g__Butyrivibrio
Unclassified g__Cardiobacterium
s__anginosus
Unclassified g__Oribacterium
s__influenzae
f__Gemellaceae_Other
Unclassified o__Clostridiales
Unclassified g__Mogibacterium
f__Enterococcaceae_Other
s__durum
Unclassified f__Leptotrichiaceae
Unclassified g__Corynebacterium
Unclassified g__Granulicatella
s__tannerae
Unclassified g__Porphyromonas
Unclassified f__Neisseriaceae
Unclassified f__Gemellaceae
Unclassified g__Campylobacter
Unclassified g__Atopobium
Unclassified g__Streptococcus
Unclassified g__Veillonella
s__parvula
s__ochracea
Unclassified g__Capnocytophaga
g__Neisseria_Other
Unclassified g__Lactobacillus
Unclassified f__Comamonadaceae
s__nanceiensis
s__melaninogenica
Unclassified g__Abiotrophia
Unclassified g__Peptostreptococcus
s__pallens
s__endodontalis
Unclassified g__Catonella
Unclassified f__Lachnospiraceae
Unclassified g__[Prevotella]
Unclassified c__BD1-5
Unclassified g__Megasphaera
Unclassified g__Dialister
Unclassified g__Fusobacterium
Unclassified g__Treponema
Unclassified p__SR1
Unclassified g__Haemophilus
Unclassified f__Peptostreptococcaceae
Unclassified o__CW040
Unclassified o__Bacteroidales
Unassigned_Other
0.0
28.6
Mean proportion (%)
−6
−4
−2
0
2
4
Difference in mean proportions (%)

Supplementary Figure S3. The bacterial taxonomic abundance of all species in ASD and NUC samples; this figure was produced using STAMP.
