# Supplementary material for: Potential Associations Among Alteration of Salivary miRNAs, Saliva Microbiome Structure, and Cognitive Impairments in Autistic Children
Source: Int J Mol Sci. 2020 Aug 27;21(17):6203. doi: 10.3390/ijms21176203 (PMC7504581; doi:10.3390/ijms21176203)
Supplement: Supplementary file 1 [file ijms-21-06203-s001.zip › Supplementary Table S1.pdf]

Potential associations among alteration of salivary miRNAs, saliva microbiome structure and cognitive impairments in autistic children

Marco Ragusa[1,2§], Maria Santagati[3§], Federica Mirabella[1§], Giovanni Lauretta[1], Matilde Cirnigliaro[1], Duilia Brex[1], Cristina Barbagallo[1], Carla Noemi Domini[4], Mariangela Gulisano[4], Rita Barone[4], Laura Trovato[3], Salvatore Oliveri[3], Gino Mongelli[3,5], Ambra Spitale[3], Davide Barbagallo[1], Cinzia Di Pietro[1†], Stefania Stefani[3†], Renata Rizzo[4†], Michele Purrello[1*].

	White's non parametric t-test	Mann-Whitney-Kruskal-Wallis test	T-test/Anova
	**P-values**	**P-values**	**P-values**
**g__Filifactor**	9.99E-04	0.029812	0.0016537
**g__Rothia**	0.011	0.030216	0.012415
**g__Actinobacillus**	0.016	0.41668	0.016316
**f_weeksellaceae**	0.016	0.038872	0.011177
**g__Ralstonia**	0.023	0.046734	0.078283
**f_ pasteurellaceae**	0.029	0.021476	0.122916
**g__Aggregatibacter**	0.035	0.16022	0.020473
**g__Tannerella**	0.044	0.038368	0.041844
**c_TM7**	0.045	0.027238	0.015501
**g__Moryella**	0.045	0.027239	0.033352

Supplementary Table S1. Statistical analysis of the bacterial abundance at the genus level in ASD and NUC groups by a two-sided White's non-parametric t-test, Mann-Whitney-Kruskal-Wallis test and T-test/Anova, p-value <0.05.
